# Analysis of *luciferase* dsRNA production during baculovirus infection of Hi5 cells: RNA hairpins expressed by very late promoters do not trigger gene silencing

**DOI:** 10.3389/finsc.2022.959077

**Published:** 2022-07-22

**Authors:** Anna Kolliopoulou, Dimitrios Kontogiannatos, Aleksander Józef Mazurek, Izabela Prifti, Vasiliki-Maria Christopoulou, Vassiliki Labropoulou, Luc Swevers

**Affiliations:** ^1^ Insect Molecular Genetics and Biotechnology, Institute of Biosciences and Applications, National Centre for Scientific Research “Demokritos”, Athens, Greece; ^2^ Department of Biomedical Sciences, University of West Attica, Athens, Greece

**Keywords:** baculovirus expression vector system, dsRNA, hairpin RNA, dsRNA-binding protein, RNAi, MS2 phage capsid protein, polyhedrin promoter

## Abstract

The baculovirus expression vector system (BEVS) has become an important platform for the expression of recombinant proteins and is especially useful for the production of large protein complexes such as virus-like particles (VLPs). An important application for VLPs is their use as vehicles for targeted delivery of drugs or toxins which requires the development of methods for efficient loading with the intended cargo. Our research intends to employ the BEVS for the production of VLPs for the delivery of insecticidal dsRNA molecules to targeted insect pests (as “dsRNA-VLPs”). A convenient strategy would be the co-expression of long dsRNAs with viral capsid proteins and their simultaneous encapsulation during VLP assembly but the capacity of the BEVS for the production of long dsRNA has not been assessed so far. In this study, the efficiency of production of long RNA hairpins targeting the *luciferase* gene (“dsLuc”) by the *polyhedrin* promoter during baculovirus infection was evaluated. However, RNAi reporter assays could not detect significant amounts of dsLuc in Hi5 cells infected with recombinant baculovirus, even in the presence of co-expressed dsRNA-binding protein B2-GFP or the employment of the MS2-MCP system. Nevertheless, dot blot analyses using anti-dsRNA antibody revealed that baculovirus-mediated expression of B2-GFP resulted in significant increases in dsRNA levels in infected cells that may correspond to hybridized complementary viral transcripts. Using B2-GFP as a genetically encoded sensor, dsRNA foci were detected in the nuclei that partially co-localized with DAPI staining, consistent with their localization at the virogenic stroma. Co-localization experiments with the baculovirus proteins vp39, Ac93, ODV-E25 and gp64 indicated limited overlap between B2-GFP and the ring zone compartment where assembly of nucleocapsids and virions occurs. Stability experiments showed that exogenous dsRNA is resistant to degradation in extracts of non-infected and infected Hi5 cells and it is proposed that strong unwinding activity at the virogenic stroma in the infected nuclei may neutralize the annealing of complementary RNA strands and block the production of long dsRNAs. Because the strong stability of exogenous dsRNA, transfection can be explored as an alternative method for delivery of cargo for dsRNA-VLPs during their assembly in baculovirus-infected Hi5 cells.

## Introduction

The baculovirus expression vector system (BEVS) has become one of the most powerful and versatile platforms for the production of recombinant proteins in large quantities (1). As eukaryotic expression system, it accommodates extensive post-translational modifications such as glycosylation and supports formation of multi-subunit protein complexes (2, 3). Recent applications of the BEVS include the expression of large protein complexes for structural analysis (4), the production of gene therapy vectors and vaccines (5, 6) and the transduction of mammalian cells (7, 8). Importantly, expression of membrane and secreted proteins could be improved following appropriate engineering of the host cells (9–11), which was facilitated by the application of gene silencing technologies such as CRISPR-Cas and RNAi (12, 13). The generation of recombinant baculoviruses for protein expression has become increasingly straightforward, particularly with the commercialized Bac-to-Bac™ system that uses transformed bacteria maintaining baculovirus genomes as large plasmids (“bacmids”) into which foreign genes can be inserted from intermediary pFastBac vectors by targeted transposition (14). The Bac-to-Bac™ BEVS utilizes the prototype baculovirus species Autographa californica multiple nucleopolyhedrovirus (AcMNPV) in combination with permissive lepidopteran cell lines derived from *Spodoptera frugiperda* (Sf9, Sf21) or *Trichoplusia ni* (Hi5) (15).

The baculovirus life cycle is complex and can be divided in three phases: early, late and very late (16). The transition from early to late is marked by the initiation of viral DNA replication and expression of the baculovirus-encoded viral RNA polymerase for transcription of late viral genes. During the late phase, budded viruses (BV) are generated that are responsible for spreading of the infection in cell culture. Unique to baculoviruses is the very late stage of infection during which occlusion-derived viruses (ODV) are produced. ODVs accumulate in the nuclei of the infected cells and, in the case of wild-type AcMNPV, become occluded in large polyhedra or occlusion bodies. The BEVS technology is based on the employment of the very powerful very late promoters such as *polyhedrin* and *p10* to drive the expression of recombinant proteins to extraordinarily high levels (17).

In our research, the BEVS is employed to produce virus-like particles (VLPs) for their use as carriers of dsRNA to cause gene silencing in targeted insect pests (18, 19). For this purpose, VLPs from dsRNA viruses such as *Bombyx mori* Cypovirus 1 (BmCPV-1; *Reoviridae*) are considered as suitable vehicles since they naturally encapsulate dsRNA genomes and are known to infect the midgut of the lepidopteran insect *B. mori* (20). When the *polyhedrin* promoter was used to express the capsid shell protein (CSP; also known as VP1) of BmCPV-1 by the BEVS, spontaneous assembly of VLPs of 50-70 nm size was observed, which is similar to authentic BmCPV-1 virions (21–23). However, to be effective, VLPs need to be loaded with dsRNA cargo, which seems to be most easily accomplished when dsRNA, in the form of RNA hairpins, becomes co-expressed with CSP in the BEVS.

While protein expression with the BEVS is very efficient, much less is known with respect to its capability to produce dsRNA. The capacity to produce large amounts of nucleic acids during infection must be high since many baculovirus genome copies accumulate in the nuclei of the infected cells during the very late stages and the large amounts of protein synthesis naturally require sufficient amounts of mRNA, tRNA and ribosomes (16). Despite these considerations, it remains to be tested whether the BEVS can be used for the production of specific long dsRNA molecules at equivalent levels to recombinant proteins when their expression is driven by very late promoters.

In this study, the capacity of the BEVS to produce specific long dsRNA molecules by the *polyhedrin* promoter is investigated. However, while the presence of dsRNA-binding proteins and loss of *Dcr-2* increased the content of dsRNA in the infected cells, it did not result in the synthesis of significant amounts of dsLuc. Since RNA hairpin production could not be achieved in the BEVS, loading of VLPs with dsRNA therefore may require the addition of exogenous dsRNA during the assembly process. Surprisingly, exogenous dsRNA molecules were shown to have remarkable stability in extracts of lepidopteran cell lines, revealing that this strategy may be feasible.

## Materials and methods

### Construction of recombinant baculoviruses

The intermediary vectors pFastBac-1 and pFastBac-Dual were used for the generation of recombinant baculoviruses by the Bac-to-Bac™ baculovirus expression system (Thermo Fisher Scientific). Expression of the RNA hairpin corresponding to the *luciferase* ORF (dsLuc) was carried out by the *polyhedrin* promoter as engineered in the pFastBac-1 or pFastBac-Dual plasmids. The dsLuc hairpin consists of a 398 bp stem separated by a loop of 118 nt (**Supplementary Figure S1**). In a control construct, a direct (antisense) repeat of the Luc sequence (asLuc) was also cloned downstream of the *polyhedrin* promoter (**Supplementary Figure S2**). DsRNA-binding proteins such as the B2 RNAi suppressor protein of Flock house virus (FHV) (24), the fluorescent dsRNA-binding protein B2-GFP (25) and the tandem dimer of MS2 coat protein fused to GFP (tdMCP-GFP) (amplified from plasmid phage-ubc-nls-ha-tdMCP-gfp; obtained from Addgene; 26) were expressed by the *p10* promoter as engineered in the pFastBac-Dual vector. GFP was expressed either by the *polyhedrin* promoter (pFastBac-1 vector) or by the *p10* promoter (pFastBac-Dual vector). In all cases, recombinant proteins were expressed with a Myc-His tag at the C-terminus (27) for detection of expression by anti-Myc antibodies. A 219 bp fragment containing four recognition sites for tdMCP-GFP (MS2-binding sites or MBSs) (amplified from plasmid pUbC-FLAG-24xSuntagV4-oxEBFP-AID-baUTR1-24xMS2V5-Wpre; obtained from Addgene; 28) was also cloned downstream of dsLuc in pFastBac-Dual vectors that co-express the dMCP-GFP (see below).

To generate recombinant bacmid genomes by Tn7 transposition, DH10B *Escherichia coli* bacteria containing AcMNPV bacmid DNA and the Tn7 transposition helper plasmid pMON7124 were transformed with pFastBac-1 or pFastBac-Dual vector derivatives as described (29). Recombinant bacmids were selected on LB-agar plates containing 50 μg/ml kanamycin, 10 μg/ml tetracyclin and 7 μg/ml gentamycin by blue/white selection as described (Bac-to-Bac baculovirus expression system manual, ThermoFisher Scientific).

**Figure 1:**
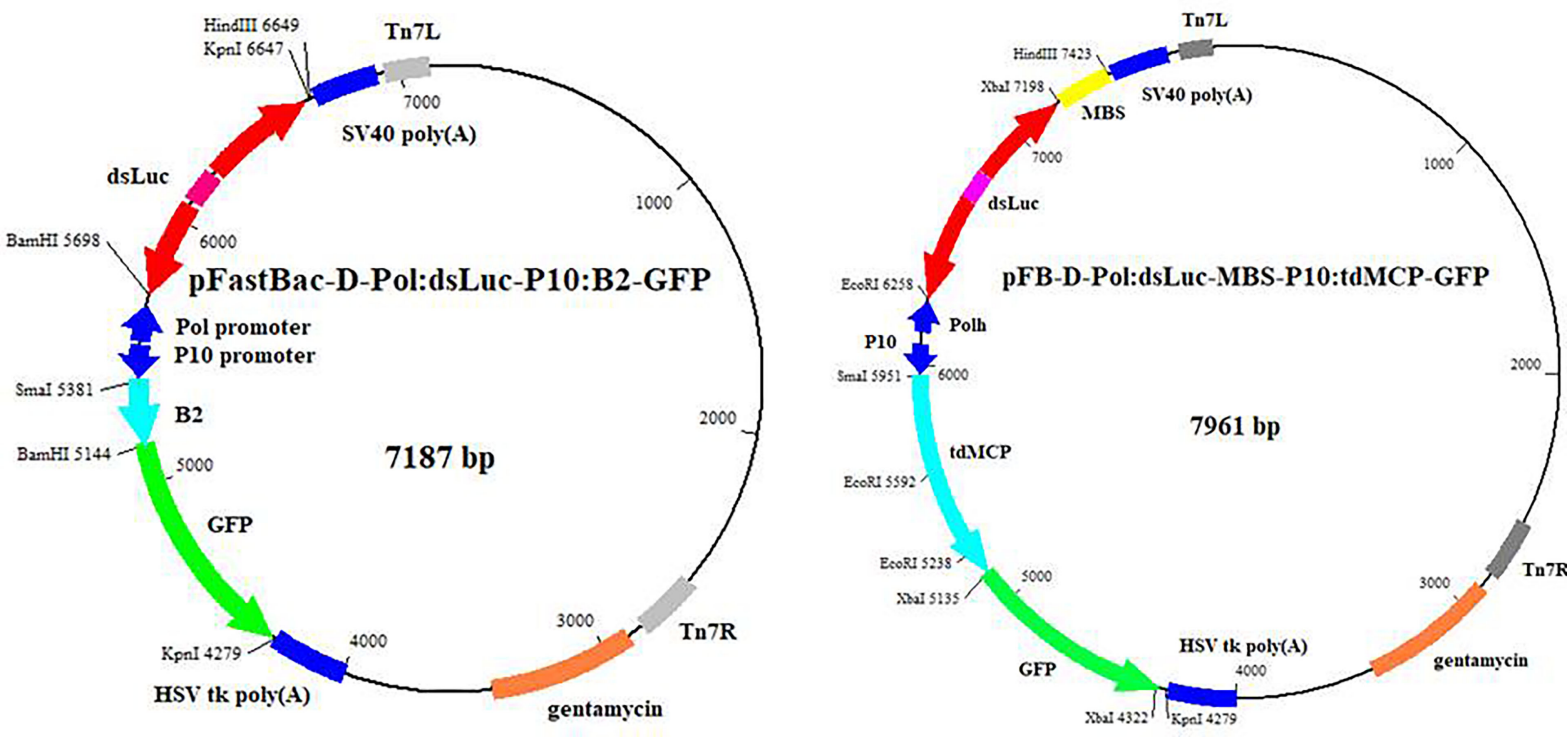
Genetic maps of the pFastBac-Dual vectors that were used to generate the recombinant baculoviruses AcMNPV-dsLuc/B2-GFP and AcMNPV-dsLuc-MBS/tdMCP-GFP. All other pFastBac-Dual vectors have a similar basic organization: production of auxiliary proteins (GFP, B2, B2-GFP, tdMCP-GFP) by the p10 promoter and synthesis of RNA molecules (dsRNA, asRNA, MBS) by the polyhedrin promoter. A summary of all pFastBac vectors is presented in **Table 1**.

An overview of the recombinant baculoviruses used in this study is presented in **Table 1**. Genetic maps of the pFastBac-Dual vectors that express dsLuc in combination with B2-GFP or tdMCP-GFP are shown in the **Figure 1**. It is noted that in the pFastBac-Dual vector that co-expresses tdMCP-GFP by the *p10* promoter, the dsLuc sequence downstream of the *polyhedrin* promoter is followed by a 0.2 kb fragment that contains four MBS; see **Figure 1**; 28).

**Table 1:** Overview of recombinant baculoviruses used in this study. All recombinant proteins were expressed with Myc-His tag at the C-terminus.

Recombinant AcMNPV	pFastBac vector	*polyhedrin* promoter insert	*p10* promoter insert
AcMNPV-dsLuc	pFastBac-1	dsLuc RNA hairpin	–
AcMNPV-GFP	pFastBac-1	GFP	–
AcMNPV-B2	pFastBac-1	B2	–
AcMNPV-dsLuc/GFP	pFastBac-Dual	dsLuc RNA hairpin	GFP
AcMNPV-dsLuc/B2	pFastBac-Dual	dsLuc RNA hairpin	B2
AcMNPV-B2-GFP	pFastBac-Dual	–	B2-GFP
AcMNPV-dsLuc/B2-GFP	pFastBac-Dual	dsLuc RNA hairpin	B2-GFP
AcMNPV-asLuc/B2-GFP	pFastBac-Dual	asLuc RNA antisense	B2-GFP
AcMNPV-tdMCP-GFP	pFastBac-Dual	–	tdMCP-GFP
AcMNPV-dsLuc/tdMCP-GFP	pFastBac-Dual	dsLuc RNA hairpin	tdMCP-GFP
AcMNPV-dsLuc-MBS/tdMCP-GFP	pFastBac-Dual	dsLuc RNA hairpin + 4 x MBS	tdMCP-GFP
AcMNPV-asLuc/tdMCP-GFP	pFastBac-Dual	asLuc RNA antisense	tdMCP-GFP
AcMNPV-asLuc-MBS/tdMCP-GFP	pFastBac-Dual	asLuc RNA antisense + 4 x MBS	tdMCP-GFP

*MBS, MS2-binding site.*

### Cell culture and generation of recombinant baculoviruses

Hi5 and Sf21 tissue culture cells were maintained in IPL-41 medium containing 10% fetal calf serum as previously described (24). Recombinant bacmid DNA was isolated from DH10B host bacteria by the lysozyme method and 0.5-2.5 μg of total DNA was used to transfect Hi5 host cells using Escort IV (Sigma) according to established methods (29). Supernatants from transfected cells were collected 7-10 days after transfection and used to re-infect fresh host cells (30). Supernatants of infected cell cultures, obtained 7 days after infection, were stored at -70°C as high titer (> 10^9^ pfu/ml) stocks of recombinant infectious baculovirus particles.

### Time course of infection of Hi5 cells with recombinant baculovirus

Hi5 cells (500,000 cells/mL) were infected with recombinant baculovirus at multiplicity of infection (m.o.i.) of 5. The progression of infection was monitored using a Zeiss Axiovert 25 inverted microscope equipped for both bright-field and fluorescence illumination. Samples were collected at different time points (1 day, 3 days, 7 days) for further biochemical analysis. Cells were separated from the culture medium after centrifugation (500g, 5 min) and cell pellets were re-suspended in phosphate-buffered saline (PBS; 100 μL per 500.000 cells). Re-suspended cell pellets were incubated at -20 °C for 30 min and subsequently centrifuged (12000g, 15 min) to obtain both soluble (supernatant) and insoluble (pellet) cell fractions.

### Construction of expression vectors for red fluorescent protein (RFP)-fusion proteins

RFP and ORFs of baculovirus genes were amplified with Phusion polymerase (ThermoFisher Scientific) using primers that were engineered to contain *Bam*HI or *Bgl*II restriction sites to maintain the ORFs in the fusions (**Table 2**). Protein expression was carried out by the pEA-MycHis vector (27). All fusions contained the RFP at the C-terminus and were tagged with the MycHis sequence. Transfection of Hi5 cells with the RFP-fusion expression vectors and the pEA-Dicer-2-MH vector (31) together with the pIE-1 plasmid was carried out using Escort IV as transfection agent as described (32).

**Table 2:** PCR primers used in this study.

Gene	Primers	Fragment size
	**Detection of recombinant baculovirus genes** **and *Dicer-2***	
*he65* (AcMNPV)	Fw: ATCGCATGCACGTTTCTAAA Rev: GCGAGTATCAACAAGAATGG	390 bp
*egt* (AcMNPV)	Fw: GCTACGGTTTGGCGGAAAAC Rev: ACGGCGCGCTCTTTACAAGA	330 bp
*gp64* (AcMNPV)	Fw: AGCTTGTTGATGTGCGCAT Rev: ATAAAAGCTGCGTGTCTGCT	320 bp
dsLuc stem	Fw: AATTCAGCGGGGGCCACCTG Rev: GAAGTCGGGGAAGCGGTTGC	398 bp
GFP	Fw: AGGGCGATGCCACCTACGGC Rev: CTTCAGCTCGATGCGGTTCA	278 bp
B2	Fw: ATGCCAAGCAAACTCGCGCTAA Rev: GGCCTTTCCCTCTAGGTATGCC	219 bp
B2-GFP	Fw: ATGCCAAGCAAACTCGCGCT Rev: CTTCAGCTCGATGCGGTTCA	621 bp
MS2-binding site	Fw: ACCGGTAACCTACAAACGGG Rev: TCGTTTGACGTATATTGCAC	219 bp
*Dicer-2* (dsRNA-binding domain)	Fw: CCGTATGGAGGTCGCCTAAC Rev: GCACTCTTCCCTTAGTCCCG	1548 bp
*Dicer-2* (PAZ-domain)	Fw: TCGAGCAAGTTTTAGCCATTGC Rev: CTGTGATGCGGACACTAGCT	1630 bp
	**Construction of RFP-fusion proteins**	
RFP	Fw: ATGGATCC *GGTGGA*ATGgcctcctccgaggac Rev: ATAGATCTCGTTTCTCGTTCAGCTTTTTTG	735 bp
N-terminus *odv-e25* (AcMNPV)	Fw: ATGGATCC *CAAC*ATGTGGGGAATCGTGTTAC Rev: ATAGATCTATTGAAATTTAATGCATTCG	79 bp
*odv-e25* (AcMNPV)	Fw: ATGGATCC *CAAC*ATGTGGGGAATCGTGTTAC Rev: ATAGATCTATTCATTTCTCTGTATGATTTAT	800 bp
*ac93* (AcMNPV)	ATGGATCC *CAAC*ATGGCGACTAGCAAAACGATC ATAGATCTATTTACAATTTCAATTCCAATGAG	487 bp
*gp64* (AcMNPV)	Fw: ATAGATCT *CAAC*ATGCTACTAGTAAATCAGTCAC Rev: ATAGATCTATATTGTCTATTACGGTTTCTAA	1606 bp

Primers for construction of RFP-fusion proteins were engineered with *Bam*HI or *Bgl*II restriction sites to maintain the ORF. RFP was also extended by two Gly residues at the N-terminus (italics in the primer sequence).

### Knock-out of *Dcr-2* gene in Hi5 cells with CRISPR-Cas

A search for candidate protospacer-associated motif (PAM) sequences (NGG) in the ORF of the *Dcr-2* gene of *Trichoplusia ni* (XM_026878152.1) was performed by use of crispr.dbcls.jp/doc online tool, according to the following parameters: to promote RNA stability, GC content of target-sequence should represent 40-80% of total sequence; to avoid off-target effect, the sequence should be 17-24 nt long; cloned sequence should start with a G. Then the 5’ start site of target sequence was determined 20 nucleotides upstream of PAM. Off-targets were excluded by use of BLAST tool in the NCBI database.

For the CRISPR/Cas9-mediated gene editing experiments in insect cells, pCas9 plasmids were kindly provided by Dr. D.L. Jarvis (University of Wyoming, WY, USA). The pCas9 plasmids contained a) *Streptococcus pyogenes Cas9* endonuclease gene ORF under AcMNPV *ie1* promoter, b) two *Sap*I restriction sites for insertion of the target sequence between TnU6 promoter and guide sgRNA, and c) puromycin resistance gene (*pac*) under AcMNPV *hr5* enhancer sequence and promoter elements of *ie1* (12). To create *Sap*I compatible ends on the selected sequences, specific trinucleotides (ATT or AAC) were added at the 5’ end of the sequence of forward or reverse complement direction, respectively. Complementary sequences of the designed oligonucleotides were allowed to anneal at 95°C for 5 min and slowly cool down, phosphorylated and then ligated to dephosphorylated *Sap*I-digested pCas9 vector (33). Positive clones were screened by PCR and selected clones were confirmed by sequencing.

To achieve maximal levels of knock-out, two to four pCas9 plasmids each targeting one of two different regions of the ORF (or both) in the *Dicer-2* gene were simultaneously transfected to Hi5 cells. Different domains in the ORF (dsRNA-binding or PAZ) were each targeted by two separate guide RNAs at sequences separated by 100-150 bp (**Table 3**). In experiments targeting a single region with two guide RNAs, Hi5 cells were transfected with 300ng of each pCas9 plasmid (600 ng in total) using Escort IV as transfection agent. In experiments that employed all guide RNAs and targeted two different regions, 150 ng of each pCas9 plasmid (600 ng in total) was used in transfection. Knock-out of *Dicer-2* was verified by PCR using primers that flanked the targeted region (**Table 2**).

**Table 3:** Guide RNA sequences for knock-out of *Dicer-2* in Hi5 cells.

Start position	Strand	Sequence	GC %	Target domain
1923	+	GCCCATCGCTTGCCCTATTAAGG	55.00	dsRNA-binding
2044	–	CCCTTGACGATGCTGCCAATGCG	55.00	dsRNA-binding
2647	–	CCGCCGTATGAGGACAGGATTAA	45.00	PAZ
2773	–	CCTCAGTCTCATTTCGATTCAGA	40.00	PAZ

### PCR

For detection of baculovirus genome copies, insoluble cell fractions were extracted with phenol/chloroform and 1 μL amounts of the water fractions were used for PCR. For detection of *Dcr-2*, total nucleic acid extracts from cells were prepared by urea/SDS lysis buffer (34). PCR was carried out by *Taq* polymerase (EnzyQuest, Heraklion, Crete) and amplification conditions were 94°C, 30 s; 55°C, 30 s; 72°C, 30 s to 2 min for 30 or 35 cycles. Primer pairs were specific for baculovirus genes (*he65*, *egt*), dsLuc stem, B2, GFP, B2-GFP and regions in the ORF of *Dcr-2* (**Table 2**).

### Western blot

Protein samples were prepared in cracking buffer and processed for western blot analysis as described before (32). Expression of MycHis-tagged protein was carried out by the monoclonal anti-Myc antibody (1:1000 dilution; Cell Signaling). Detection of the Vp39 capsid protein of AcMNPV was carried out by a specific monoclonal antibody (1:100 dilution; kindly provided by Dr. Taro Ohkawa, University of California, Berkeley; 35).

### DsRNA dot blot

Five μL aliquots of cellular soluble extracts (PBS) or insoluble extracts (after phenol/chloroform extraction), corresponding to approximately 50,000 cells, were spotted on a positively charged nylon membrane (Roche Diagnostics GmbH) and left to dry. In parallel, solutions of positive control Luc dsRNA were also applied. After cross-linking with UV (1 min, 312nm, 15 W; Vilber Loumat UV transilluminator), membranes were processed as described for western blot. After blocking for 1 hr in PBS containing 0.1% Tween-20 (PBS-T) and 5% non-fat milk, membranes were incubated with the J2 anti-dsRNA IgG2a mouse monoclonal antibody (SCICONS, Nordic MUbio) diluted 1:1000 in PBS-T with 5% non-fat milk overnight at 4°C. Visualization of dsRNAs was achieved by standard protocols using an HRP-coupled anti-mouse antibody (Chemicon) at 1:1000. Pierce SuperSignal West Pico chemiluminescent substrate (Thermo Fisher Scientific) was used for detection in an ImageQuant™ LAS 4000 (GE Healthcare) imaging system. To produce positive control Luc dsRNA, the RNA hairpin of *luciferase* that was used for expression in recombinant baculovirus (**Supplementary Figure S1**) was subcloned in the pLitmus 38i vector (New England Biolabs). After linearization of the plasmid, dsLuc RNA hairpin was produced by T7 RNA polymerase (New England Biolabs) as described (31). Densitometry of dot blot signals was carried out by ImageJ software (36). Statistical analysis (paired t-test) was carried out by GraphPad Prism 4 software.

### Northwestern blot

Nucleic acid extracts (20 μg, obtained by phenol extraction and isopropanol precipitation) of Hi5 cells that were harvested at 4 days post infection (dpi) were loaded on 5% polyacrylamide gel in Tris-Borate-EDTA (TBE) buffer as described (25). Polyacrylamide gel electrophoresis was carried out in TBE buffer for 20 hours at 20V at 4°C. Electrophoresis was visualized by ethidium bromide (EtBr; Sigma) staining. Electrophoresed RNAs were blotted onto a Hybond^®^-N+ nylon membrane (Amersham) by wet electroblotting in 3 mM NaOH for 2 hours at 110 V. Efficient transfer was confirmed by EtBr staining. RNAs were fixed in a Vilber Lourmat UV transilluminator (312 nm, 15 W tubes) for 5 minutes. Membranes were subsequently processed for western blot using the J2 anti-dsRNA mouse monoclonal antibody (SCICONS, Nordic MUbio) diluted at 1:1000 as primary antibody as described above.

### Immunofluorescence

Transfected and infected cells were seeded on poly-L-Lysine-coated slides (Sigma), fixed with 4% formaldehyde in PBS for 10 min and permeabilized with PBS containing 0.1% Triton X-100 for 5 min. After blocking with 3% BSA in PBS for 1 hour at room temperature, cells intended to be used for immunolocalization experiments were treated overnight at 4°C with monoclonal antibodies anti-Vp39 or anti-Myc (Cell Signaling) at 1:100 in PBS containing 1% BSA. After five washes with PBS, samples were incubated with CF^®^568-conjugated goat anti-mouse secondary antibody (Biotium) at 1:100 in PBS containing 1% BSA for 1 hr at room temperature. Following five washes with PBS and staining with DAPI (0.5 μg/mL, 5 min), cells were mounted in Mowiol 4-88 (Sigma) for observation by fluorescence microscopy.

### Fluorescence *in situ* hybridization (FISH)

Hi5 cells were grown on sterile cover slips in 6-well plates and attached cells were infected with AcMNPV-dsLuc/B2-GFP or AcMNPV-dsLuc-MBS/tdMCP-GFP viruses at m.o.i. 5 for 4 days. Uninfected cells were used as negative controls. Sense and anti-sense probes were designed against the single stranded loop of the dsLuc construct (118 nt; **Supplementary Figure S1**) which was cloned in the pLITMUS 38i vector (NEB). Sense and anti-sense RNAs of *luciferase* fragment were *in vitro* transcribed by T7 RNA polymerase and labeled with the Alexa Fluor^®^ 594 reactive dye (red) using the FISH Tag™ RNA Multicolor Kit (Thermo Scientific) according to manufacturer’s instructions. Cover slips were removed from the 6-well plates and subjected to hybridization using a hybridization buffer containing 40% v/v formamide (Sigma), 10% w/v dextran sulfate (Sigma), 1 x Denhardt’s solution (0.02% w/v Ficoll, 0.02% w/v polyvinylpyrrolidone, 0.2 mg/ml bovine serum albumin (BSA); Sigma), 4 x saline-sodium citrate (SSC) buffer (0.6 M sodium chloride, 60 mM trisodium citrate, pH 7), 10 mM dithiothreitol, 1 mg/ml yeast t-RNA (Sigma), 1 mg/ml denatured and sheared salmon sperm DNA (Sigma) and 1 μg/ml Alexa Fluor^®^ 594 labeled sense or anti-sense probes. Hybridization was performed overnight in a 50°C water bath. Post-hybridization, cover slips were washed successively in 2 x and 1 x SSC twice at 37°C and unbound ssRNA probe was removed by incubating with 20 μg/ml RNase A (Sigma) in NTE buffer (500 mM NaCl, 10 mM Tris, 1 mM EDTA, pH 8.0). For co-localization with baculovirus-expressed (MycHis-tagged) B2-GFP or tdMCP-GFP proteins, cover slips were washed with PBS for three times and subjected to immunofluorescence as described above. Anti-Myc and Alexa Fluor 488^®^-labeled anti-mouse were used as primary and secondary antibodies (both at 1/100), respectively. After washing, the cells were stained with DAPI (0.5 μg/ml) (Sigma) and mounted with anti-bleaching solution (FISH Tag™ RNA Multicolor Kit, Thermo Scientific).

### Confocal microscopy

For the experiments involving immunolocalization and subcellular localization by use of fluorescence, a TCS SP8 MP (Leica, Germany) microscope equipped with HC Plan APO 63x, N.A. 1.40 oil immersion objective was used. Images were analyzed with ImageJ.

### RNAi reporter assay

Hi5 cells (500,000 cells per well in a 24-well plate) were transfected with ecdysone reporter genes pERE-Luc (150 ng) and pERE-GFP (50 ng) together with RNA of infected cells (up to 500 ng) (31). One day after transfection, tebufenozide (ecdysone agonist) was added at 200 nM to induce reporter expression and cells were collected at 48 hrs for GFP (normalization) and luciferase (targeted by *luciferase* RNA hairpin) measurements. RNA was isolated from both soluble and insoluble cellular extracts by Nucleozol (Machery-Nagel, Germany). Total nucleic acid extracts were treated with RNase-free DNase I (1 U/μg for 15 min; TaKaRa) before Nucleozol extraction. As positive control, *in vitro* transcribed *luciferase* RNA hairpin was co-transfected at 1 to 10 ng quantities. Non-specific dsRNA (dsmalE) was used as negative control (31). GraphPad Prism 4 software was used for statistical analysis (unpaired t-test).

## Results

### Expression of B2 dsRNA-binding protein, but not expression of dsRNA Luc hairpin, results in higher production of dsRNA

To evaluate the capacity of RNA hairpin constructs for the production of long dsRNA molecules during baculovirus infection, four different recombinant baculoviruses were constructed. AcMNPV-GFP represents wild-type baculovirus infection in the absence of expression of RNA hairpin (the presence of GFP is included to monitor progression of infection). AcMNPV-dsLuc/GFP evaluates the production of RNA hairpin in the absence of dsRNA-binding protein while AcMNPV-dsLuc/B2-GFP was used to investigate whether co-expression of the dsRNA-binding protein B2 of FHV could result in higher levels of dsLuc during infection. Finally, AcMNPV-asLuc/B2-GFP that expresses a direct antisense repeat of the *luciferase* fragment (asLuc) was used to evaluate the impact of B2-GFP on dsRNA accumulation in the absence of expression of a long RNA hairpin by the polyhedrin promoter.

**Figure 2:**
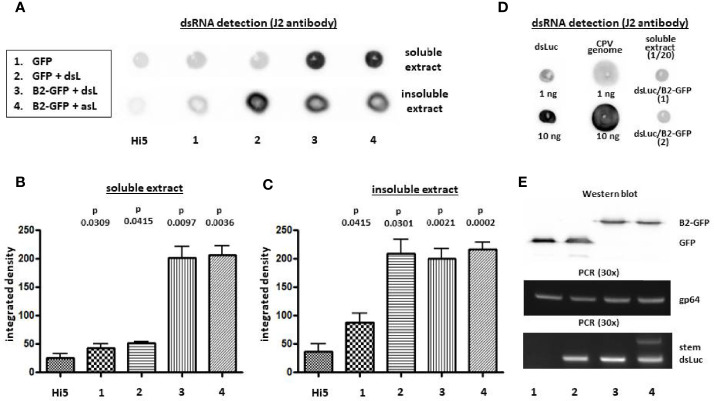
Dot blot analysis of dsRNA accumulation in Hi5 cells that were infected with the recombinant baculoviruses AcMNPV-GFP (1), AcMNPV-dsLuc/GFP (2), AcMNPV-dsLuc/B2-GFP (3) and AcMNPV-asLuc/B2-GFP (4) at 3 days post infection (dpi). **(A)** displays a representative dot blot of both soluble and insoluble cellular extracts. **(B, C)** present the quantification of dot blot signals following repeat experimentation (N =3) for soluble and insoluble extracts, respectively. P values indicate the significance for the comparison with uninfected Hi5 cells (paired t-test). **D** compares the signal of 1 and 10 ng of *in vitro* transcribed dsLuc or dsRNA genome of *Bombyx mori* Cypovirus (CPV) with the signal of 1/20 of the soluble extract of Hi5 cells infected with AcMNPV-dsLuc/B2-GFP (corresponding to 25,000 cells; 2 repeats), as indicated. **(E)** shows recombinant protein expression [GFP (30 kDa) and B2-GFP (39 kDa)] in the cellular media by western blot and the accumulation of baculovirus genomes and dsLuc in the insoluble cellular extracts [PCR detection of *gp64* (320 bp) and dsLuc stem sequence (398 bp)]. The exposure time for the dot blot in **(A–D)** is 5 minutes and 1 minute, respectively. dsL, dsLuc; asL, antisense Luc.

After infection of Hi5 cells with each baculovirus, cellular extracts of infected cells at 3 dpi were analyzed for dsRNA production by dot blot analysis using J2 anti-dsRNA antibody, which is specific to double-stranded regions of RNA and does not cross-react with double-stranded DNA (37). The dot blot assay allowed us to reliably detect amounts of *in vitro* transcribed *luciferase* hairpin (dsLuc) and other dsRNA molecules as low as 1 ng (**Figure 2D**). Using this technique, it was demonstrated, first, that dsRNA levels increase during baculovirus infection compared to uninfected cells, and, second, that co-expression of B2-GFP results in the accumulation of dsRNA to relatively high levels (**Figure 2A**). In the soluble cell extracts, however, expression of dsLuc did not result in higher amounts of dsRNA accumulation (compare AcMNPV-GFP with AcMNPV-dsLuc/GFP and AcMNPV-asLuc/B2-GFP with AcMNPV-dsLuc/B2-GFP; **Figures 2A, B**). In insoluble extracts, considerably higher amounts of dsRNA were observed after infection with AcMNPV-dsLuc/GFP compared with AcMNPV-GFP while no differences were observed between AcMNPV-dsLuc/B2-GFP and AcMNPV-asLuc/B2-GFP (**Figures 2A, C**). Finally, it should be noticed that dsRNA levels in all extracts remained relatively low and were estimated at less than 1 ng per 25,000 cells (**Figure 2D**). The expression of dsLuc or B2-GFP did not seem to affect the course of baculovirus infection since equivalent amounts of viral DNA or recombinant protein (GFP or B2-GFP) were observed in all conditions (**Figure 2E**).

### Absence of gene silencing activity in extracts of cells infected with baculoviruses that express dsLuc

**Figure 3:**
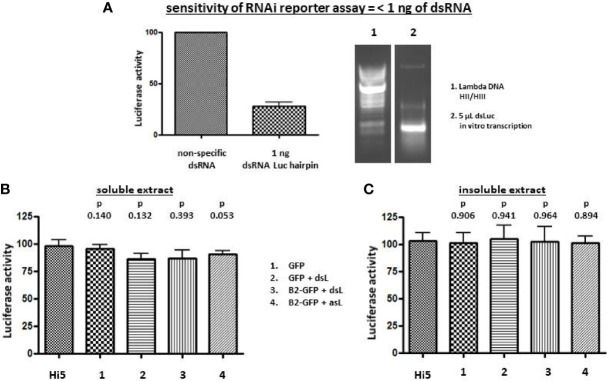
Luciferase silencing assay using extracts of Hi5 cells that were either uninfected or infected with the recombinant baculoviruses AcMNPV-GFP (1), AcMNPV-dsLuc/GFP (2), AcMNPV-dsLuc/B2-GFP (3) and AcMNPV-asLuc/B2-GFP (4) at 3 days post infection (dpi). **(A)** shows silencing of the *luciferase* reporter by 1 ng of *in vitro* transcribed dsLuc (N = 5). A picture of an ethidium bromide-stained gel that shows *in vitro* transcribed dsLuc is shown at the right in lane 2. Lane 1 shows the molecular weight marker (Lambda DNA digested with HincII and HindIII). **(B, C)** present the luciferase activity following co-transfection with 250 ng of RNA from soluble and insoluble cellular extracts, respectively. Luciferase activity was normalized to samples from uninfected cells that were transfected with non-specific dsRNA (31). N = 3. Abbreviations: dsL = dsLuc; asL = antisense Luc; HII/HIII = digested with HincII and HindIII.

While overall dsRNA production significantly increased during baculovirus infection (**Figure 2**) and the stem sequence of dsLuc could readily be amplified by PCR (**Figure 2E**), the amount of dsRNA corresponding to dsLuc in extracts from cells infected with AcMNPV-dsLuc derivatives remained to be established. A previously developed RNAi reporter assay, based on the silencing of a *luciferase* gene by specific dsRNA (31), was employed for this purpose. When the dsLuc hairpin was subjected to *in vitro* transcription by T7 RNA polymerase, efficient production of dsRNA was observed in a single reaction, indicating robust hybridization of the inverted repeat into a double-stranded structure (**Figure 3A**). The *in vitro* produced hairpin exhibited potent silencing activity in the RNAi reporter assay since quantities of 1 ng could silence the *luciferase* reporter to 28 ± 9% (N=5) compared to non-specific dsRNA (set at 100%). When RNA extracts (prepared with Nucleozol and free of protein) from Hi5 cells infected with AcMNPV-dsLuc were tested, however, no silencing activity was observed (**Figures 3A, B**). The increase in dsRNA levels by co-expression of B2-GFP (**Figure 2**) did not result in an increase in silencing activity (**Figures 3A, B**). Both soluble and insoluble RNA extracts of infected cells were devoid of measurable *luciferase* silencing activity despite the presence of the dsLuc hairpin expression cassette in the recombinant baculoviruses.

### Knock-down/knock-out of *Dcr-2* in host cells does not result in a significant increase in dsRNA production during baculovirus infection

Because co-expression of B2-GFP resulted in higher amounts of dsRNA in baculovirus-infected cells (**Figure 2**), the effect of knock-out of *Dicer-2* in the host cells was also investigated. To achieve silencing of *Dicer-2*, a CRISPR/Cas9 expression vector was employed that is capable to achieve efficient silencing in Hi5 cells (11). For maximal effect, essential domains in the ORF of *Dicer-2* (dsRNA-binding and PAZ) were targeted with two guide RNAs at sites separated by 100-150 bp (**Supplementary Figure S3**). The simultaneous targeting of both domains by co-transfection of four pCas9 plasmids was also attempted. In all cases, the *Dicer-2* gene was severely disrupted 4 days after transfection since no or only weak signals were detected after PCR amplification of genomic DNA using primers that flank the targeted sites (**Supplementary Figure S3**). Even when a single region was targeted, disruption of the other region was also observed (**Supplementary Figure S3**). When *Dicer-2* knock-out cells were infected with AcMNPV-GFP or AcMNPV-dsLuc/GFP, the accumulation of GFP fluorescence and viral genomic DNA occurred with similar kinetics as in control cells (**Supplementary Figure S3**). Measurement of dsRNA levels in the soluble cellular extracts indicated a minor increase in *Dicer-2* knock-out cells infected with AcMNPV-dsLuc/GFP at 6 dpi (**Supplementary Figure S3**). The effect was clearly more limited than the approximately 4-fold increase in dsRNA levels following co-expression of B2-GFP by the *p10* promoter in baculovirus-infected cells (**Figure 2**).

### Time course of dsRNA production during baculovirus infection using genetically encoded B2-GFP

**Figure 4:**
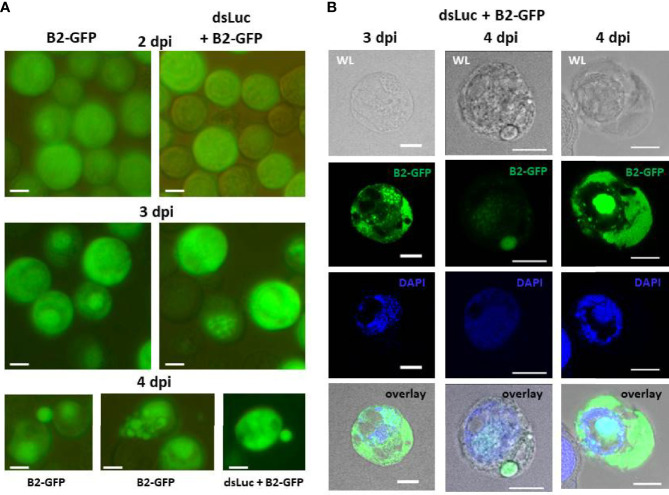
Accumulation of B2-GFP fluorescence in Hi5 cells infected with AcMNPV-B2-GFP or AcMNPV-dsLuc/B2-GFP. **(A)**: B2-GFP fluorescence in live cells detected by conventional inverted fluorescence microscope at 2, 3 and 4 days post infection (dpi). **(B)**: Confocal images of selected Hi5 cells infected with AcMNPV-dsLuc/B2-GFP at 3 and 4 dpi. Shown are white light (WL), GFP, DAPI and overlay (WL-GFP-DAPI) images. Note that in the right sample, green fluorescence in the nucleus is over-exposed to reveal the weaker signals in the cytoplasm. At lower intensity, the signal in the nucleus shows distinct foci as observed in the two other samples. The white bar in the photographs corresponds to a length of 10 μm.

At 2 dpi, fluorescence in Hi5 cells was mostly diffuse although some weak fluorescent foci could sometimes be observed (**Figure 4A**). The presence of fluorescent foci in distinct areas became much stronger after 3 days. At 4 dpi, some cells disintegrated and large cell fragments with intense fluorescence could often be observed scattered among the infected cells (**Figure 4**). Overall, the pattern of fluorescent foci formation was not different between cells infected with AcMNPV-B2-GFP and AcMNPV-dsLuc/B2-GFP (**Figure 1**).

The subcellular pattern of B2-GFP fluorescence at 3 and 4 dpi was also investigated in more detail by confocal microscopy (**Figure 4B**). The distinct fluorescent foci overlapped with DAPI staining of DNA in the nuclei, while the staining in the cytoplasm was diffuse and restricted to defined areas and sometimes to large round vesicles (**Figure 4B**). Individual cells could differ considerably with respect to staining pattern and intensity of fluorescence.

Finally, fluorescent foci in the nuclei that overlapped with DAPI staining and more diffuse and compartmentalized staining in the cytoplasm was also observed after infection of Sf21 cells with AcMNPV-B2-GFP, both in the absence and in the presence of dsLuc (**Supplementary Figure S4**).

### Localization of B2-GFP foci in nuclear domains of baculovirus-infected cells

To understand better the pattern of dsRNA accumulation during infection, B2-GFP fluorescence was compared with the distribution of viral proteins that are components of BVs or ODVs, or are involved in the trafficking of virions during infection. To visualize the baculovirus proteins, expression vectors for fusion proteins with RFP were generated which were transfected to Hi5 cells immediately before infection with AcMNPV-dsLuc/B2-GFP. This strategy has been used previously to study the subcellular localization of baculovirus proteins during infection (38, 39).

The subcellular distribution of the following baculovirus proteins were compared with B2-GFP: (1) ODV-E25, which is an ODV envelope protein that also associates with BVs in AcMNPV (40, 41); (2) Ac93, which associates with endosomal sorting complex required for transport (ESCRT)-III proteins at the nuclear membrane (42); and (3) gp64, which encodes the envelope fusion protein of BVs required for infectivity (43). For ODV-E25, two constructs were tested, which gave very similar results: employing either the full-length protein (ODV-E25) or the N-terminal 25 AA (N-ODV-E25) (40).

When initially studied in the absence of baculovirus infection, transfection of expression plasmids resulted in robust production of fusion proteins in Hi5 cells (**Supplementary Figure S5**). While non-fused RFP accumulated mainly in the soluble fraction during western blot analysis, all viral protein-RFP fusions were mostly detected in the insoluble fraction, indicating their altered subcellular distribution (**Supplementary Figure S5**).

**Figure 5:**
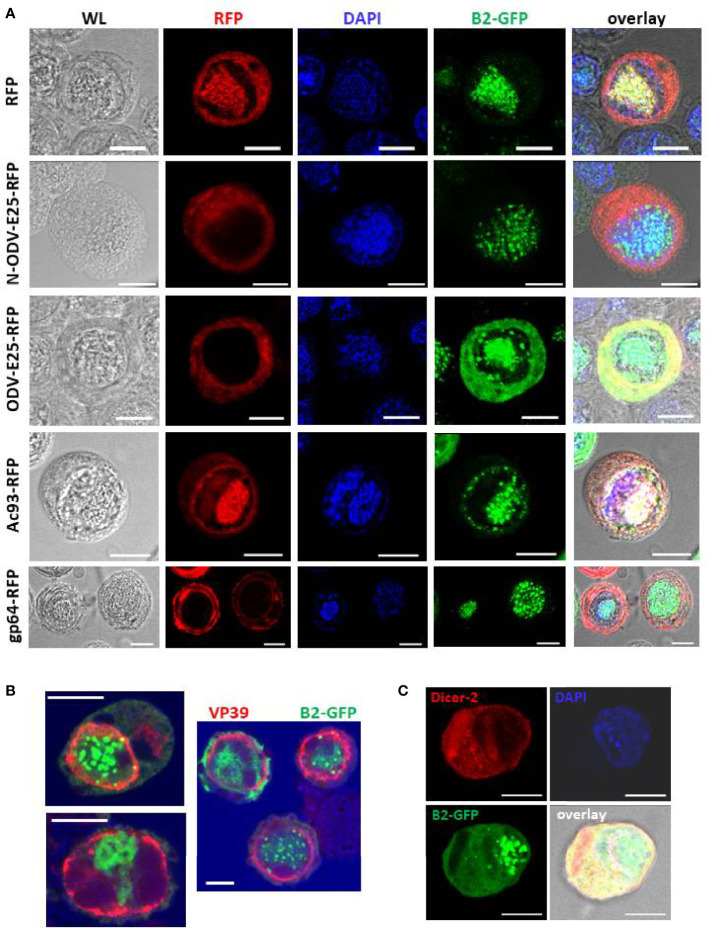
Subcellular localization of foci of B2-GFP fluorescence (detection of dsRNA) together with selected baculovirus proteins at 3 dpi. **Panel (A)**: Double staining for B2-GFP and baculovirus proteins that were expressed as C-terminal fusions with RFP as indicated. **Panel (B)**: Comparison of B2-GFP fluorescence with immunostaining of VP39. **Panel (C)**: Comparison of B2-GFP fluorescence with immunostaining of Dicer-2 following transfection of an expression plasmid of MycHis-tagged Dicer-2. Abbreviations: WL, white light. Overlay refers to a composite image of all channels. The white bar in the photographs corresponds to a length of 10 μm.

In uninfected cells, non-fused RFP localizes to the cytoplasm (**Supplementary Figure S6**). At 3 dpi, however, RFP has an additional nuclear localization that coincides with B2-GFP and partially overlaps with DAPI (**Figure 5A**). The expansion of RFP localization may be related to the increased permeability of the nuclear membrane during infection (44); it also indicates nuclear subdomains that are not accessible to protein accumulation (“empty zones” in the nucleus that also do not show fluorescence by DAPI or B2-GFP) (**Figure 5A**, top row).

N-ODV-E25- and ODV-E25-RFP showed similar cytoplasmic localization in both uninfected and infected cells (**Supplementary Figure S6** and **Figure 5A** **¸**2^nd^ and 3^rd^ row). In infected cells, the fluorescence may reflect its association with BVs during their egress since ODV-E25 has been found to be associated with both BVs and ODVs (41). Diffuse B2-GFP fluorescence strongly co-localizes with ODV-E25 in the cytoplasm (**Figure 5A**, 3^rd^ row).

Ac93-RFP fluorescence overlapped with GFP but showed much higher intensity in the nucleus of infected cells (overlapping with DAPI) and additionally displayed a ring that followed the shape of the nucleus (**Figure 5A**), consistent with its role in nuclear membrane remodeling for ODV assembly (44). Interestingly, this ring zone also shows foci of B2-GFP (**Figure 5A**, 4^th^ row) besides the robust co-localization with DAPI in the center of the nucleus (virogenic stroma).

Fluorescence of gp64-RFP was localized in the cytoplasm, as expected (**Supplementary Figure S6** and **Figure 5A**). At 3 dpi, increased intensity was observed around the nucleus and at the plasmamembrane (**Figure 5A**, bottom row). Also in these cells, B2-GFP fluorescence partially co-localized with DAPI in the center of the nucleus.

The relative localization of B2-GFP with the major capsid protein, VP39, was also investigated (**Figure 5B**). Immunofluorescence using a specific antibody detected VP39 concentrated in a ring zone in the nuclei of the infected cells, as reported previously (45). By contrast, B2-GFP foci were located centrally in the nuclei where they co-localize with DAPI in the virogenic stroma (**Figures 5A, B**). Occasionally, a focal point of B2-GFP overlapped with the ring zone of VP39 distribution (**Figure 5B**).

Finally, immunostaining of Dicer-2 was performed after transfection of an expression vector of MycHis-tagged Dicer-2 in Hi5 cells before infection with AcMNPV-dsLuc/B2-GFP. In uninfected cells, Dicer-2 localizes mainly to the cytoplasm (31) and this pattern was also observed at 3 dpi (**Figure 5C**). No obvious co-localization was observed between Dicer-2 and B2-GFP foci in the nucleus.

### Failure of the MS2-MCP system to sequester dsRNA *luciferase* hairpins in baculovirus-infected cells

In an attempt to prevent the possible loss of *luciferase* RNA hairpin from the infected cells, the MS2-MCP system was employed that is used to visualize mRNA trafficking in live cells and to purify RNAs from cellular extracts (46, 47). The MS2-MCP system is based on the selective binding of stem loops, characterized as MBSs, by a homodimer of the MS2 coat protein (48). In the pFastBac-Dual vector, a fragment containing four MBSs was cloned downstream from the dsLuc hairpin in the *polyhedrin* expression cassette (**Figure 1**). After transcription, dsLuc RNA was therefore fused with four stem-loops that have high affinity for the MCP dimer. The *p10* module, on the other hand, was engineered to express a tandem dimer of MCP (tdMCP) that assembles as an antiparallel dimer following expression (48). In addition, tdMCP contained nuclear localization signals to confine the localization to the nuclei of infected cells and was fused to GFP for visualization of expression (as tdMCP-GFP).

Four different baculoviruses were used to evaluate the capacity of the MS2-MCP system to sequester dsLuc in infected cells. All baculoviruses express tdMCP-GFP from the *p10* promoter but express from the *polyhedrin* promoter either *luciferase* RNA hairpin (dsLuc) with or without MBS (AcMNPV-dsLuc-MBS/tdMCP-GFP or AcMNPV-dsLuc/tdMCP-GFP), or an antisense direct repeat of the *luciferase* fragment (asLuc) with or without MBS (AcMNPV-asLuc-MBS/tdMCP-GFP or AcMNPV-asLuc/tdMCP-GFP).

**Figure 6:**
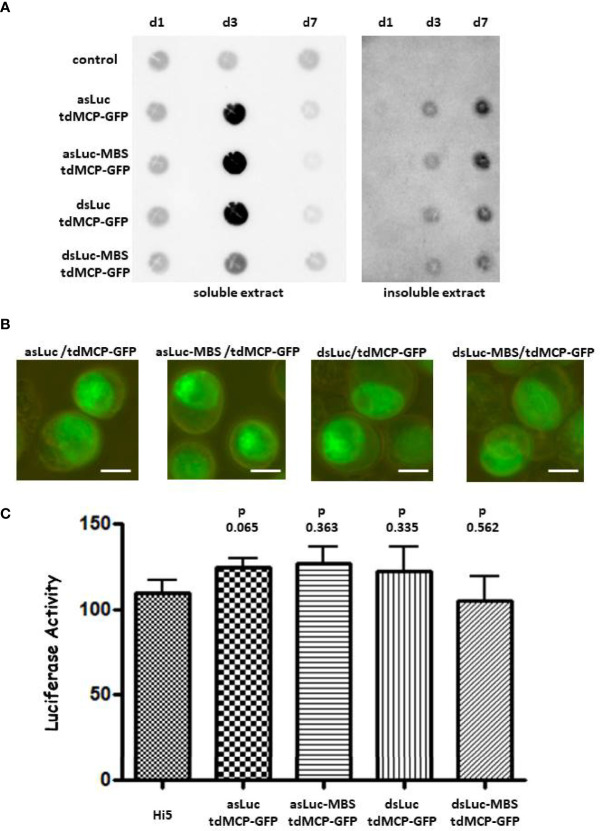
Accumulation of dsRNA in Hi5 cells that were infected with the recombinant baculoviruses AcMNPV-asLuc/tdMCP-GFP, AcMNPV-asLuc-MBS/tdMCP-GFP, AcMNPV-dsLuc/tdMCP-GFP or AcMNPV-dsLuc-MBS/tdMCP-GFP. Abbreviations: d1, d3 and d7 refer to the days of infection. **(A)**: Dot blot analysis using J2 anti-dsRNA antibody of soluble and insoluble extracts corresponding to approximately 50.000 uninfected (control) and infected Hi5 cells as indicated. **(B)**: Fluorescence microscope images of expression of tdMCP-GFP in the nuclei of infected cells. The size of the bar corresponds to 10 μm. Panel **(C)**: Luciferase silencing assay using extracts of Hi5 cells that were either uninfected or infected with the recombinant baculoviruses. Bars present the luciferase activity following co-transfection with 500 ng of RNA from soluble cellular extracts. Luciferase activity was normalized to samples from uninfected cells transfected with non-specific dsRNA (31). N = 3.

Infections of Hi5 cells by the different baculoviruses were analyzed at 1, 3 and 7 dpi by PCR and western blot. PCR detected a similar accumulation of baculovirus DNA in all infection conditions although a delay was observed for the infection with AcMNPV-dsLuc-MBS/tdMCP-GFP (**Supplementary Figure S7**). Relatively low amounts of tdMCP-GFP and VP39 protein also accumulated in the cellular medium after infection with AcMNPV-dsLuc-MBS/tdMCP-GFP (**Supplementary Figure S7**). When dsRNA accumulation was measured by dot blot analysis using J2 anti-dsRNA antibody, significant increases were observed for all baculoviruses at 3 dpi in soluble extracts that were reduced to much lower levels at 7 dpi, reflecting cell lysis at this very late stage of infection (**Figure 6A**). In insoluble extracts, dsRNA levels continued to increase between 3 and 7 dpi, although the levels were lower than in the soluble extracts (**Figure 6A**).

Immunofluorescence showed the accumulation of tdMCP-GFP primarily in the nuclei of the infected cells, both as a diffuse signal and by the appearance of foci (**Figure 6B**). Foci of tdMCP-GFP were more apparent in infections with AcMNPV expressing asLuc and were independent of the presence of MBS in the constructs. When RNA from soluble extracts (500 ng) was tested in RNAi reporter assays, no silencing of *luciferase* activity was observed for any condition of the MS2-MCP system (N = 3; **Figure 6C**).

### Analysis of different dsRNA species in infected cells

**Figure 7:**
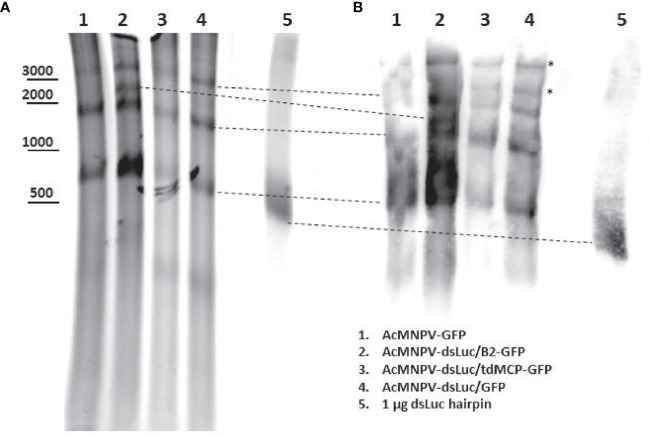
Northwestern blot analysis of different dsRNA species in Hi5 cells infected with different recombinant baculoviruses at 4 days post infection (dpi) as indicated. **(A)**: Ethidium bromide-stained non-denaturing polyacrylamide gel. The approximate mobility of DNA markers is indicated. **(B)**: Western blot using anti-dsRNA J2 antibody. The correspondence of the bands detected by western blot with those detected with ethidium bromide staining is indicated with dashed lines. Additional bands in western blot are indicated by asterisk. DsLuc RNA (produced by *in vitro* transcription) approximately corresponds to 0.4 kb dsRNA with a loop of 0.1 kb.

To further investigate the variety of dsRNA types that are produced during infection, 20 μg of nucleic acid extract from cells infected with different recombinant baculoviruses was separated in a 5% non-denaturing polyacrylamide gel. Ethidium bromide staining revealed different major bands of which the molecular weight ranged from 0.5 kb to more than 3 kb (**Figure 7A**). Some of the bands correspond to structural RNAs (e.g. ribosomal 18S and 28S species of 1.9 kb and 4.1 kb ssRNA, respectively) also detected in uninfected cells. However, unique bands were observed after baculovirus infection (**Supplementary Figure S8**). After transfer to positively charged nylon membrane and probing with anti-dsRNA J2 antibody, the bands detected with ethidium bromide stained positive with the J2 antibody while also additional dsRNA bands of high MW appeared (**Figure 7B**). A band of approximately 0.5 kb was detected with J2 antibody in extracts from infected cells but this band also appeared in infections with baculovirus that does not express dsLuc (AcMNPV-GFP; lane 1 in **Figure 7B**) and therefore does not correspond to the *luciferase* RNA hairpin.

**Figure 8:**
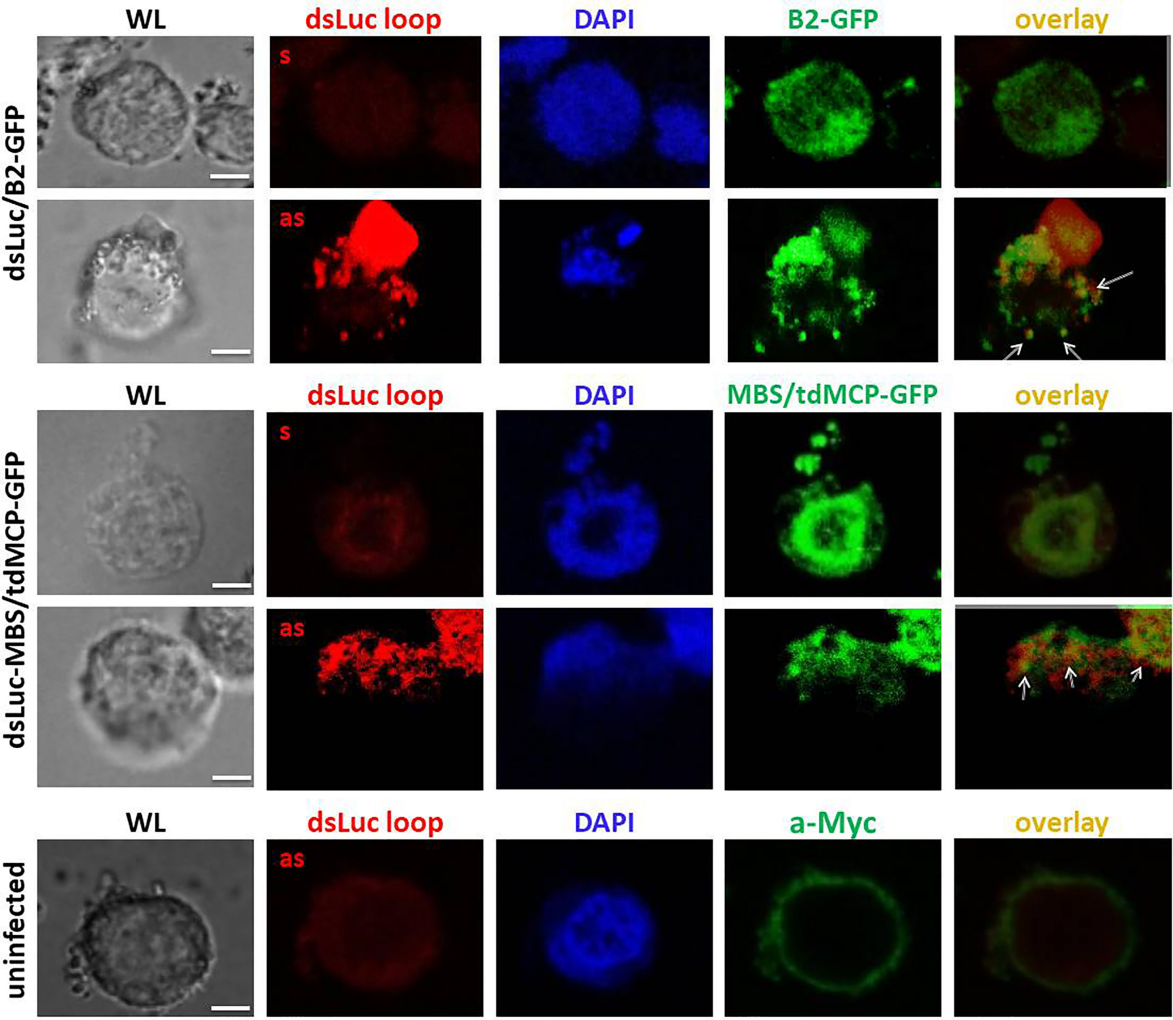
Fluorescence *in situ* hybridization for detection of the loop of dsLuc in Hi5 cells infected with the recombinant baculoviruses AcMNPV-dsLuc/B2-GFP and AcMNPV-dsLuc-MBS/tdMCP-GFP viruses, as indicated, at 4 days post infection (dpi). Double staining of the proteins B2-GFP and tdMCP-GFP was also performed by immunostaining using anti-Myc antibody. Shown are white light (WL), Alexa Fluor^®^ 594 (sense or antisense loop), Alexa Fluor^®^ 488 (B2-GFP or tdMCP-GFP), DAPI and Alexa Fluor^®^ 594/Alexa Fluor^®^ 488 overlay images. Overlap of signals between FISH and immunostaining are indicated by arrows. FISH signals obtained with the antisense probe showed much higher intensity compared with the sense probe in the infected cells. There was only limited overlap between RNA hairpin loop detection (FISH) and the localization of dsRNA (detection of B2-GFP and tdMCP-GFP). The white bar in the photographs corresponds to a length of 10 μm.

To further evaluate the expression of dsLuc in infected cells, FISH experiments were performed. Because the stem sequence of dsLuc is expressed as dsRNA, it cannot be probed unless denaturation is performed, which requires more harsh conditions than for DNA because of the higher stability of dsRNA (49). It was therefore decided to use as a probe the loop sequence of the dsLuc hairpin (118 nt; **Supplementary Figure S1**) which was transcribed as sense or antisense RNA by T7 RNA polymerase. Because the loop sequence is transcribed as ssRNA during AcMNPV-dsLuc infection, it can be hybridized with high efficiency with antisense RNA probes. In addition, co-staining for B2-GFP or tdMCP-GFP would reveal the localization of dsRNA molecules. Much stronger FISH signals were obtained with the antisense probe than with the sense probe for both AcMNPV-dsLuc/B2-GFP and AcMNPV-dsLuc-MBS/tdMCP-GFP infections, indicating transcription of the loop sequence (**Figure 8**). FISH signals were identified in the nucleus (detection with antisense probe in AcMNPV-dsLuc-MBS/tdMCP-GFP infection; **Figure 8**) but also in the cytoplasm as is also observed for B2-GFP (detection with antisense probe in AcMNPV-dsLuc/B2-GFP infection; **Figure 8**). Double staining with B2-GFP and tdMCP-GFP reveals only limited overlap of signal with the antisense RNA probe (**Figure 8**).

### Stability of dsLuc in uninfected and baculovirus-infected Hi5 cell extracts

One explanation for the absent or low levels of dsLuc in infected Hi5 cells could be the degradation of dsRNA by cellular or baculovirus-encoded nucleases. To investigate this possibility, pellets of Hi5 cells that were either uninfected or infected with AcMNPV-GFP or AcMNPV-B2 (3 dpi) were suspended in PBS and subjected to freeze-thawing to disrupt the integrity of the cellular structures. The obtained cellular extracts (50 μL corresponding to 500,000 cells) were spiked with 250 ng of *in vitro* transcribed dsLuc hairpin and subsequently incubated at 28°C (the temperature of cell culture maintenance) for 24 hr. Incubation of dsLuc in PBS was carried out as control. Nucleic acids from the incubated extracts were compared with extracts that were prepared immediately after the spiking with dsLuc.

**Figure 9:**
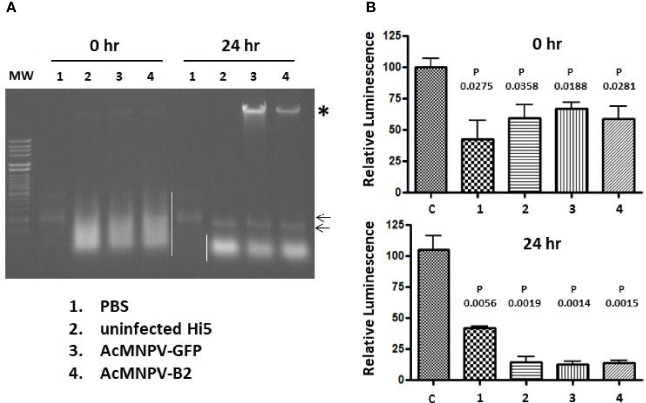
Incubation of exogenous dsRNA in PBS or in extracts from Hi5 cells that were either uninfected or infected with AcMNPV-GFP or AcMNPV-B2 for 3 days as indicated.**(A)**: Ethidium bromide-stained agarose gel. The position of dsRNA is indicated by small arrows and cellular RNAs are marked with white line. Note that the dsRNA is masked by cellular RNAs in the 0 hr extracts. Viral genomic DNA is indicated with asterisk and is enriched in the incubated extracts because of degradation of cellular RNAs. “MW” (molecular weight) corresponds to Lambda DNA digested with *Hind*III and *Hinc*II. **(B)**: Luciferase silencing assays performed with RNA (500 ng) from extracts that were prepared immediately after spiking with dsRNA (top) or from extracts that were incubated after spiking for 24 hr at 28°C (bottom). N = 3. “C” corresponds to RNA from untreated Hi5 cells. P values indicate the significance for the comparison with Hi5 cells that were transfected with control dsRNA (dsmalE). Viral genomic DNA is indicated with asterisk.

When analyzed by agarose gel electrophoresis, dsRNA showed a remarkable stability in extracts from both uninfected and infected cells during the 24 hr incubation period although a small change in mobility was observed compared with dsRNA incubated in PBS (**Figure 9A**). Comparison of the nucleic acids obtained after 0 hr and 24 hr also reveals considerable reduction of cellular RNA after 24 hr together with the appearance of high levels of viral genome DNA in the infected cells (**Figure 9A**; lanes corresponding to cellular extracts were loaded with equivalent levels of nucleic acid). When RNA extracts (500 ng; prepared with Nucleozol after treatment with DNase) were tested in the RNAi reporter assay, clear silencing of the *luciferase* gene was observed for both 0 hr and 24 hr extracts that was comparable to PBS-treated dsRNA (**Figure 9B**). Actually, more robust silencing was observed for RNA preparations after 24 hr of incubation, presumably because of the degradation of cellular RNA and the relative increase in dsRNA content. It is noted that the expression of B2 in cellular extracts was not necessary to obtain protection of dsRNA during the 24 hr incubation period (**Figure 9**; AcMNPV-B2 infection).

Because of the enrichment of exogenous dsRNA in cellular extracts that were incubated at 28°C for 24 hr, it was also decided to similarly incubate cellular extracts of Hi5 cells infected with AcMNPV-dsLuc/B2-GFP and AcMNPV-dsLuc-MBS/tdMCP-GFP (at 3 dpi) with the aim to increase the relative content of dsRNA. However, when RNA extracts were tested in the *luciferase* reporter assay, no silencing activity was detected (**Supplementary Figure S9**). Ethidium bromide-stained agarose gels of RNA from incubated extracts also did not reveal any band corresponding to dsLuc (**Supplementary Figure S9**).

## Discussion

Our research is focused on the production of genome-free VLPs from a dsRNA virus (CPV) as a vehicle for transport of dsRNA into targeted insect cells to trigger gene silencing (19). To achieve this aim, VLPs need to be loaded with dsRNA molecules which most easily can occur concomitantly with the assembly of VLPs. Since VLPs are produced by recombinant baculoviruses, it was therefore investigated to what extent the BEVS can also be used for the expression of specific long dsRNA molecules. Whether accumulation of dsRNA at high levels is feasible indeed needs to be determined and may require additional engineering, since proteins and dsRNAs have different genetic requirements, as was recently observed for a bacterial expression system (50).

In the present study, the strategy for dsRNA accumulation consisted of the expression of an RNA *luciferase* hairpin by the very late *polyhedrin* promoter of the BEVS (**Figure 1**). For detection, a sensitive RNAi luciferase reporter assay was used that could reliably sense dsRNA amounts as low as 1 ng (**Figure 3A**). However, no silencing activity was observed in RNA extracts of Hi5 cells infected with AcMNPV that express dsLuc, even in the presence of dsRNA-binding proteins such as B2-GFP that were co-expressed with dsLuc (**Figures 3B, C**). Expression levels of dsRNA at levels lower than 1 ng are too low to provide sufficient cargo for incorporation into co-expressed VLPs. Production of the icosahedral shell based on the capsid shell protein of CPV corresponds to the assembly of 120 copies of a 150 kDa protein to generate an 18 MDa protein-based VLP particle (17). Assuming the incorporation of 5 kb of dsRNA in each VLP (CPV virions can package 25 kb of dsRNA genome at high density), dsRNA with a total molecular weight of 3.2 MDa needs to be produced. Recombinant protein levels of 10 μg/mL or higher (up to 1 mg/mL) that are routinely observed with the BEVS therefore require the co-production of at least μg quantities of dsRNA to ensure efficient encapsulation.

Several hypotheses can be forwarded to explain the absent or very low levels of dsLuc accumulating in infected cells. First, it can be envisaged that dsLuc becomes encapsulated in vesicles and virions and transported outside of the infected cells. During virulent baculovirus infections, extensive blebbing at the cell surface can occur resulting in the pinching off of large parts of the cellular contents (**Figure 4**). Cell fragments that show intense fluorescence of B2-GFP regularly are observed in the cellular media during late stages of infections (**Figure 4**). In an attempt to confine dsRNAs intracellularly, dsLuc was expressed within the framework of the MS2-MCP system that was designed to restrict the presence of dsRNA in the nuclei. However, also the utilization of the MS2-MCP system did not result in the accumulation of amounts of dsLuc that could be measured by RNAi reporter assays (**Figure 6C**).

A second possibility to explain the very low amounts could be the degradation of dsLuc after its synthesis. However, incubation of total cellular extracts of Hi5 cells with exogenous dsLuc *in vitro* only revealed remarkable stability of dsRNA (**Figure 9A**). During the preparation of cellular extracts, freeze-thawing destroys cellular membranes which frees digestive enzymes from different intracellular compartments (e.g. lysosomes) to which dsLuc may become exposed. Nevertheless, exogenous dsRNA remained intact and extracts showed potent silencing activity in RNAi reporter assay (**Figure 9C**). During the incubation, levels of cellular RNAs were also significantly decreased by degradation, resulting in an increase in relative dsRNA levels. However, when this method of enrichment was applied to extracts from cells infected with baculoviruses expressing dsLuc together with dsRNA-binding proteins, again no silencing activity was detected (**Supplementary Figure S9**).

If excretion/loss or degradation of dsRNA cannot explain the absence/low levels of dsLuc production, the argument may be considered that perhaps the *luciferase* RNA hairpin with dsRNA structure is not efficiently produced during the infection. The stem of dsLuc can be easily detected by PCR (**Figure 2E**) but complete inverted repeats/hairpins are much more difficult to amplify because of their fast re-hybridization during the annealing step which results in the rapid selection of truncated products. Similarly, detection by *in situ* hybridization also faces significant difficulties. Denaturation of the strong secondary structure of RNA hairpins is required and ssRNA/DNA needs to be removed by digestion to ascertain that signals originate from dsRNA. Using a more straightforward alternative approach, the ssRNA loop of dsLuc was readily detected by FISH but co-localization with the dsRNA-binding proteins B2-GFP or tdMCP-GFP was sporadic (**Figure 8**). When RNA extracts of Hi5 cells infected with AcMNPV-dsLuc were separated by a non-denaturing polyacrylamide gel and probed with J2 antibody, several positive fragments were detected, including a fragment with a mobility similar to dsLuc (**Figure 7**). However, this fragment was also observed in infections with AcMNPV that does not express dsLuc and therefore likely corresponds to a structural RNA with double-stranded regions (see also discussion below).

An alternative explanation for the unsuccessful production of long dsRNAs during baculovirus infection relates to dsRNA unwinding activity in the nuclei of infected cells. Nucleic acid unwinding activity in the nuclei is prominent during baculovirus infection because of the unique mechanism of replication of baculovirus genomes that is proposed to occur by extensive homologous recombination (51–53). Notably, one of the baculovirus genes that promote homologous recombination, p35, also was identified as an RNAi suppressor (52, 54). Several baculovirus genes are expressed during infection, which are involved in unwinding and annealing reactions that facilitate the production of recombination intermediates (e.g. LEF-3, Ac25 and Ac42; 55–57). However, helicase unwinding activity usually is specific towards DNA or RNA substrates, although some enzymes work on both DNA and RNA (58). In addition, some DNA helicases preferentially unwind RNA-DNA hybrids, e.g. in the course of Okazaki fragment maturation during DNA replication (59). The presence of significant dsRNA unwinding activity during infection may not be surprising because of potential deleterious effects of dsRNA such as the activation of the antiviral RNAi pathway and the reduced translation efficiency of viral mRNAs that are bound by complementary sequences. DsRNA structures presumably are transiently formed since they can be detected as foci in the nucleus by B2-GFP (**Figures 4**, **5**). Our results nevertheless suggest that the regions of hybridization of complementary RNA strands (from overlapping transcripts) are rather short and do not occur for long sequences (such as dsLuc). More work is necessary to identify and characterize the viral or cellular proteins that are proposed to exhibit dsRNA unwinding activity in the nuclei of baculovirus-infected cells.

Although AcMNPV apparently cannot achieve production of significant levels of dsLuc, dot blot analysis using J2 antibody nevertheless clearly demonstrates an increase in dsRNA levels in baculovirus-infected cells compared to uninfected cells (**Figure 2**). In soluble extracts of Hi5 cells that were infected with AcMNPV that expresses GFP, dsRNA levels increase by 1.7 to 2.0-fold. Previous studies have documented the production of viral small interfering RNAs (siRNAs) of 20 nt length during baculovirus infection of lepidopteran larvae and cell lines (54, 60). Because of the high density in the baculovirus genome of genes that are transcribed from both positive and negative strands (61), plenty of opportunity exists for hybridization of complementary read-through transcripts and the emergence of dsRNA structures. In the presence of B2-GFP, much higher levels of dsRNA can be observed (approximately 8-fold increase compared to uninfected cells; **Figure 2**). Although increased levels of dsRNA may be considered as harmful for the progression of AcMNPV infection, equivalent amounts of recombinant protein nevertheless are produced by AcMNPV that express GFP or B2-GFP (**Figure 2E**), indicating similar infection kinetics.

In the literature, other reports can be found that describe infections of baculoviruses that express heterologous viral suppressors of RNAi that are derived from RNA viruses. Baculovirus expression of non-structural protein-S (NS-S) of tomato spotted wilt virus (*Tospovirus*; *Bunyaviridae*) resulted in higher viral titers and occlusion body formation as well as increased virulence (62, 63). Expression of tombovirus P19 by baculovirus promoters also increased titers of budded virus and recombinant protein production (42). By contrast, another report did not find an effect on the production of budded virus or the virulence in insects by AcMNPV that expressed B2 by the (relatively weak) inducible heat-shock promoter (64). Production of both NS-S and B2 was found to suppress dicing of exogenous substrate (62, 64), conform to their capacity to protect dsRNA from interaction with Dicer enzymes. The latter findings agree with our observations that B2-GFP over-expression results in an increase in RNA molecules with dsRNA regions in the infected cells.

The J2 antibody is reported to bind preferentially regions of dsRNA that are larger than 40 bp (65) although also interaction with shorter sequences was observed (66). Thus, the molecules that are recognized by J2 antibody may have dsRNA regions that are relatively short. Conform with this, in northwestern blot, J2 antibody recognizes bands that clearly correspond to structural RNAs such as ribosomal 18S and 28S RNA (**Figure 7**). The high amounts (> 10 μg) of ribosomal RNA that are loaded on the non-denaturing polyacrylamide gel are considered to be sufficient to be detected by the J2 antibody, presumably because of its capacity to detect short regions of dsRNA structure (65). Interestingly, new bands appear in the RNA extracts of AcMNPV-infected Hi5 cells that appear to be absent in uninfected cells (**Figure 7**). It can be speculated that these cross-reacting bands of high molecular weight may correspond to partially hybridized overlapping transcripts of positive and negative polarity from the AcMNPV genome (61). However, because of their potentially negative impact (discussion above), dsRNA structures need to be neutralized, perhaps by unwinding activity mediated by cellular and baculovirus gene products. The absence of clear bands of long dsRNA in northwestern blot with samples of infections with baculovirus contrasts strongly with the robust detection of dsRNA replication intermediates during infection with plant and insect RNA viruses (25, 65).

Based on the strong binding of the B2 protein of FHV to dsRNA, the B2-GFP fusion was developed for the live imaging of dsRNA foci in plant and insect cells infected with RNA viruses (25). Incorporation of the expression cassette for B2-GFP in the baculovirus genome resulted in high levels of green fluorescence in the infected cells (**Figure 4**). However, B2-GFP fluorescence was manifested in two patterns. First, in some cells, intense green fluorescence appeared diffuse but remained within boundaries as if sequestered by vesicular membranes (**Figure 4**). It remains to be investigated to what extent B2-GFP is bound by dsRNA when showing this pattern since it can be assumed that B2-GFP protein is expressed at much higher levels than dsRNA (B2-GFP is easily visualized in Coomassie staining while long dsRNA does not appear in ethidium bromide-stained agarose gels) (**Supplementary Figure S9**). The second pattern appears in the nuclei and constitutes fluorescent foci that are reminiscent of fluorescent cytoplasmic aggregates in cells infected with RNA virus (25). The punctuate fluorescence of B2-GFP in cells infected with RNA virus was found to co-localize with viral replication factories and therefore corresponded to the detection of replication intermediates with dsRNA structure. The dsRNA foci in the nuclei of baculovirus-infected cells most strongly, although not exclusively, co-localized with DAPI staining of DNA (**Figures 4**, **5**).

In nuclei of baculovirus-infected cells, the cellular chromatin is marginalized at the expense of an increase of the viral replication compartment or virogenic stroma (67). Double staining clearly shows areas of overlapping signals between B2-GFP and DAPI while other regions of DAPI are free from green fluorescent foci (**Figures 4B** and **5A**; **Supplementary Figure S4**). Foci of B2-GFP likely correspond to spots of high concentration of dsRNA, as can be assumed to occur in the virogenic stroma where partially complementary viral transcripts hybridize to form double-stranded regions. DAPI regions free from B2-GFP fluorescence on the other hand seem to indicate transcriptionally inactive domains such as the cellular chromatin. The staining pattern indicates that the formation of dsRNA structures is a pervasive feature of baculovirus genome transcription because of its high density in genes that are described in both positive and negative direction (61).

To further resolve the localization of B2-GFP foci, double staining experiments for localization of baculovirus proteins were performed either by fusions with RFP or by immunofluorescence using a specific antibody against VP39 (**Figure 5**). This staining revealed the occurrence of few B2-GFP foci at the viral ring zone (overlap with VP39 and Ac93-RFP fluorescence), where the envelopment of nucleocapsids occurs for the production of ODVs (44), indicating the possibility of incorporation of dsRNA into microvesicles and virions at low levels.

The analysis of dsLuc expression by recombinant AcMNPV indicates that the production of long dsRNA (RNA hairpins) is not feasible by the BEVS. High yields of protein do not seem to be compatible with high levels of dsRNA and therefore other strategies need to be explored for the efficient loading of dsRNA cargo into CPV-based VLPs. Recently, a reverse genetics system for *Dendrolimus punctatus* CPV was described in which recombinant AcMNPV was used to express T7 RNA polymerase that was capable to synthesize the RNA of the different genome fragments (68). Evidence was shown that the exogenous RNA segments could be incorporated in virions and feeding of occlusion bodies resulted in reproductive infection in larvae. The efficiency of production of dsRNA segments, however, was not documented in this study and it should be noted that only a relatively small amount of virions may be needed for successful infections.

Our experiments indicate a remarkable stability of exogenous dsRNA when added to extracts of infected and non-infected Hi5 cells (**Figure 9**). After the incubation period, dsLuc was functional in luciferase silencing reporter assays, indicating that it was also not modified in a manner that could interfere with RNAi. Although it was proposed above that long RNA hairpins may become unwound after transcription during baculovirus infections, the unwinding activity is predicted to occur only in the vicinity of the virogenic stroma. After disruption of the cellular structure (freeze-thawing of cell suspensions in PBS), the unwinding activity may become too diluted to have an impact on dsRNA stability. Thus, a strategy for incorporation of dsRNA into VLPs would consist of the pre-loading of host cells by *in vitro* synthesized dsRNA (e.g. by transfection of μg quantities) before infection with recombinant virus and VLP production.

## Data Availability Statement

The raw data supporting the conclusions of this article will be made available by the authors, without undue reservation.

## Author Contributions

AK, DK, and LS participated in the design of the study, collected and analyzed data. AM carried out experiments with the CRISPR-Cas system and IP performed localization studies of B2-GFP and baculovirus proteins. V-MC contributed in the cloning of the expression vectors. LS wrote the first draft of the manuscript. AK, DK and VL provided critical comments and revised the manuscript. All authors contributed to the article and approved the submitted version.

## Funding

This work was also partly supported by the project “A Greek Research Infrastructure for Visualizing and Monitoring Fundamental Biological Processes (BioImaging- GR)” (MIS 5002755) which is implemented under the Action “Reinforcement of the Research and Innovation Infrastructure”, funded by the Operational Programme “Competitiveness, Entrepreneurship and Innovation” (NSRF 2014-2020) and co-financed by Greece and the E. U.

## Conflict of Interest

The authors declare that the research was conducted in the absence of any commercial or financial relationships that could be construed as a potential conflict of interest.

## Publisher’s Note

All claims expressed in this article are solely those of the authors and do not necessarily represent those of their affiliated organizations, or those of the publisher, the editors and the reviewers. Any product that may be evaluated in this article, or claim that may be made by its manufacturer, is not guaranteed or endorsed by the publisher.
