# Supplementary material for: Analysis of luciferase dsRNA production during baculovirus infection of Hi5 cells: RNA hairpins expressed by very late promoters do not trigger gene silencing
Source: Front Insect Sci. 2022 Jul 22;2:959077. doi: 10.3389/finsc.2022.959077 (PMC10926400; doi:10.3389/finsc.2022.959077)
Supplement: Supplementary file 1 [file Presentation_1.pptx]

## Slide 1
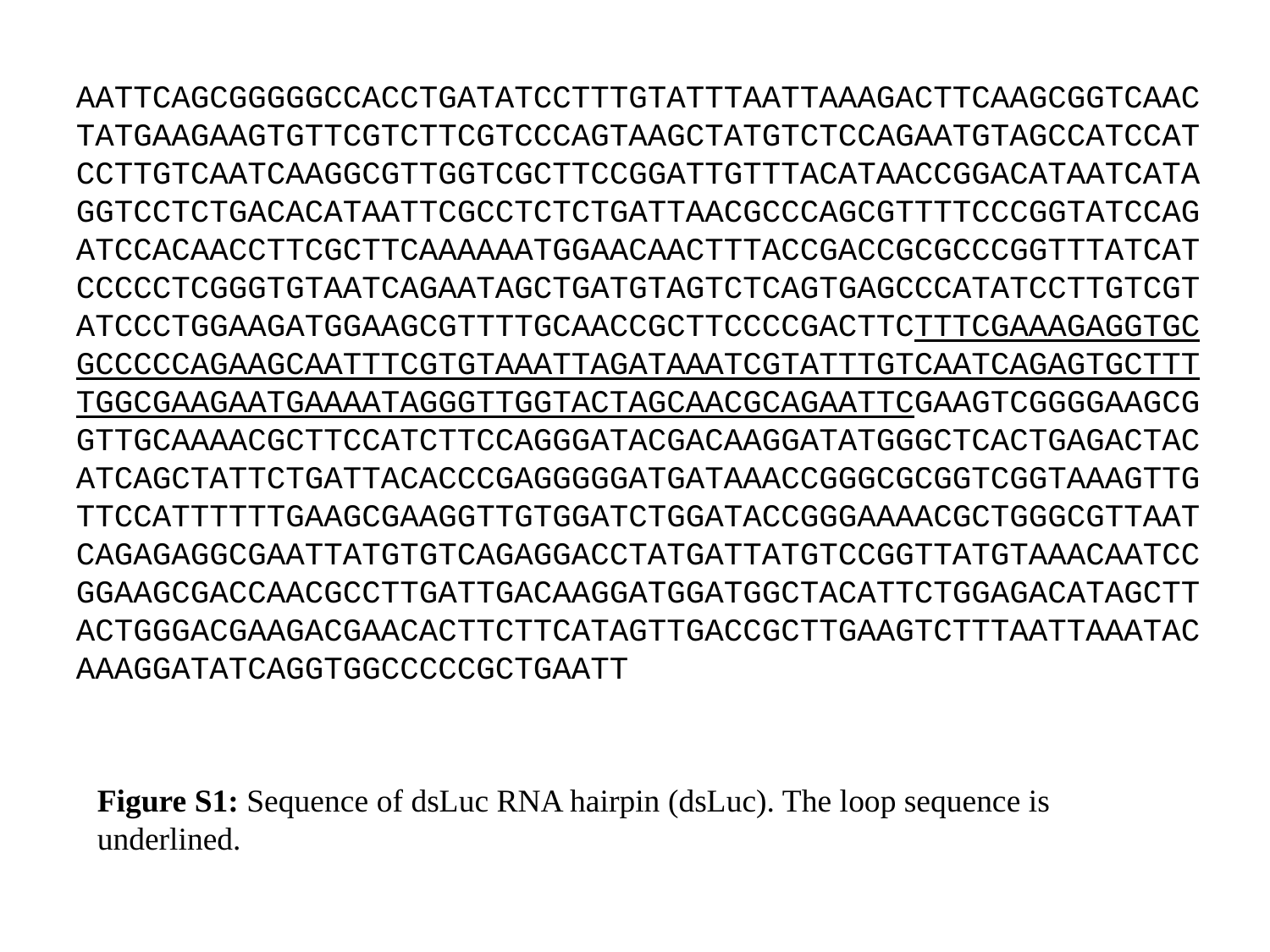

AATTCAGCGGGGGCCACCTGATATCCTTTGTATTTAATTAAAGACTTCAAGCGGTCAACTATGAAGAAGTGTTCGTCTTCGTCCCAGTAAGCTATGTCTCCAGAATGTAGCCATCCATCCTTGTCAATCAAGGCGTTGGTCGCTTCCGGATTGTTTACATAACCGGACATAATCATAGGTCCTCTGACACATAATTCGCCTCTCTGATTAACGCCCAGCGTTTTCCCGGTATCCAGATCCACAACCTTCGCTTCAAAAAATGGAACAACTTTACCGACCGCGCCCGGTTTATCATCCCCCTCGGGTGTAATCAGAATAGCTGATGTAGTCTCAGTGAGCCCATATCCTTGTCGTATCCCTGGAAGATGGAAGCGTTTTGCAACCGCTTCCCCGACTTCTTTCGAAAGAGGTGCGCCCCCAGAAGCAATTTCGTGTAAATTAGATAAATCGTATTTGTCAATCAGAGTGCTTTTGGCGAAGAATGAAAATAGGGTTGGTACTAGCAACGCAGAATTCGAAGTCGGGGAAGCGGTTGCAAAACGCTTCCATCTTCCAGGGATACGACAAGGATATGGGCTCACTGAGACTACATCAGCTATTCTGATTACACCCGAGGGGGATGATAAACCGGGCGCGGTCGGTAAAGTTGTTCCATTTTTTGAAGCGAAGGTTGTGGATCTGGATACCGGGAAAACGCTGGGCGTTAATCAGAGAGGCGAATTATGTGTCAGAGGACCTATGATTATGTCCGGTTATGTAAACAATCCGGAAGCGACCAACGCCTTGATTGACAAGGATGGATGGCTACATTCTGGAGACATAGCTTACTGGGACGAAGACGAACACTTCTTCATAGTTGACCGCTTGAAGTCTTTAATTAAATACAAAGGATATCAGGTGGCCCCCGCTGAATT
Figure S1: Sequence of dsLuc RNA hairpin (dsLuc). The loop sequence is underlined.

## Slide 2
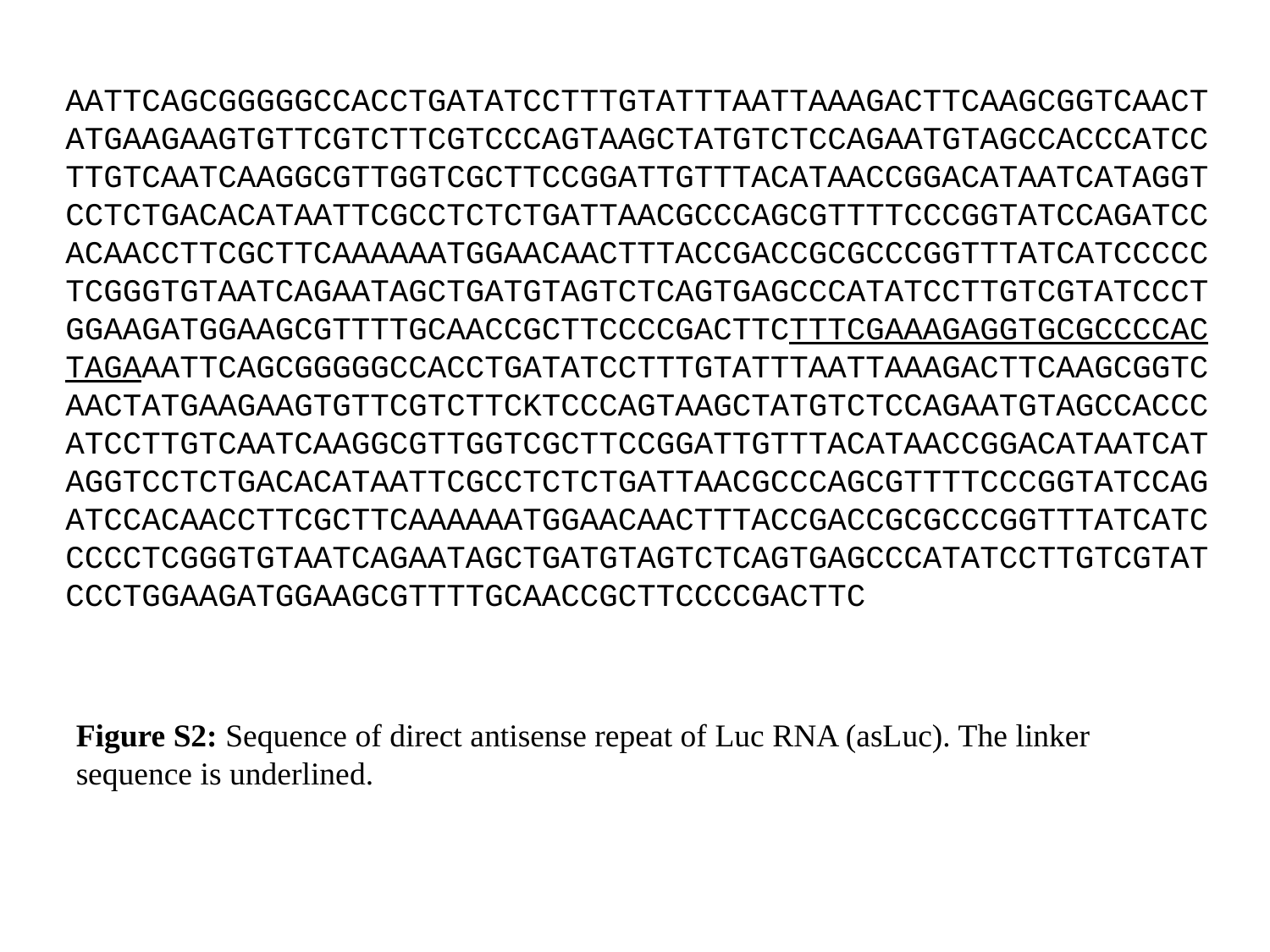

AATTCAGCGGGGGCCACCTGATATCCTTTGTATTTAATTAAAGACTTCAAGCGGTCAACTATGAAGAAGTGTTCGTCTTCGTCCCAGTAAGCTATGTCTCCAGAATGTAGCCACCCATCCTTGTCAATCAAGGCGTTGGTCGCTTCCGGATTGTTTACATAACCGGACATAATCATAGGTCCTCTGACACATAATTCGCCTCTCTGATTAACGCCCAGCGTTTTCCCGGTATCCAGATCCACAACCTTCGCTTCAAAAAATGGAACAACTTTACCGACCGCGCCCGGTTTATCATCCCCCTCGGGTGTAATCAGAATAGCTGATGTAGTCTCAGTGAGCCCATATCCTTGTCGTATCCCTGGAAGATGGAAGCGTTTTGCAACCGCTTCCCCGACTTCTTTCGAAAGAGGTGCGCCCCACTAGAAATTCAGCGGGGGCCACCTGATATCCTTTGTATTTAATTAAAGACTTCAAGCGGTCAACTATGAAGAAGTGTTCGTCTTCKTCCCAGTAAGCTATGTCTCCAGAATGTAGCCACCCATCCTTGTCAATCAAGGCGTTGGTCGCTTCCGGATTGTTTACATAACCGGACATAATCATAGGTCCTCTGACACATAATTCGCCTCTCTGATTAACGCCCAGCGTTTTCCCGGTATCCAGATCCACAACCTTCGCTTCAAAAAATGGAACAACTTTACCGACCGCGCCCGGTTTATCATCCCCCTCGGGTGTAATCAGAATAGCTGATGTAGTCTCAGTGAGCCCATATCCTTGTCGTATCCCTGGAAGATGGAAGCGTTTTGCAACCGCTTCCCCGACTTC
Figure S2: Sequence of direct antisense repeat of Luc RNA (asLuc). The linker sequence is underlined.

## Slide 3
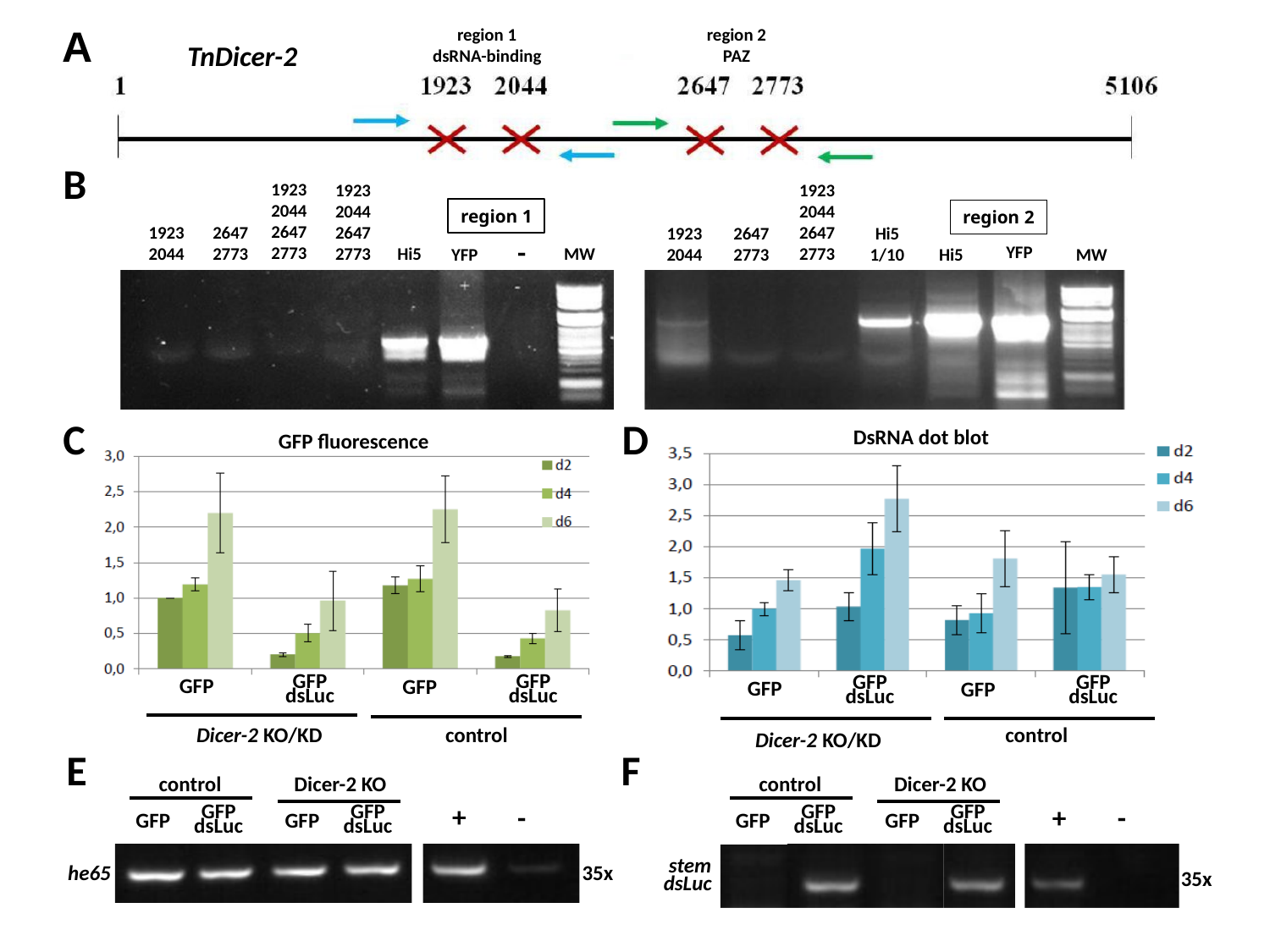

A
region 1
dsRNA-binding
region 2
PAZ
TnDicer-2
B
1923
2044
2647
2773
1923
2044
2647
2773
1923
2044
2647
2773
region 1
region 2
1923
2044
2647
2773
1923
2044
2647
2773
Hi5
1/10
-
YFP
Hi5
MW
YFP
Hi5
MW
C
D
DsRNA dot blot
GFP fluorescence
GFP
dsLuc
GFP
dsLuc
GFP
dsLuc
GFP
dsLuc
GFP
GFP
GFP
GFP
Dicer-2 KO/KD
control
control
Dicer-2 KO/KD
E
F
control
Dicer-2 KO
control
Dicer-2 KO
GFP
dsLuc
GFP
dsLuc
GFP
GFP
+
-
GFP
dsLuc
GFP
dsLuc
GFP
GFP
+
-
stem dsLuc
he65
35x
35x

## Slide 4
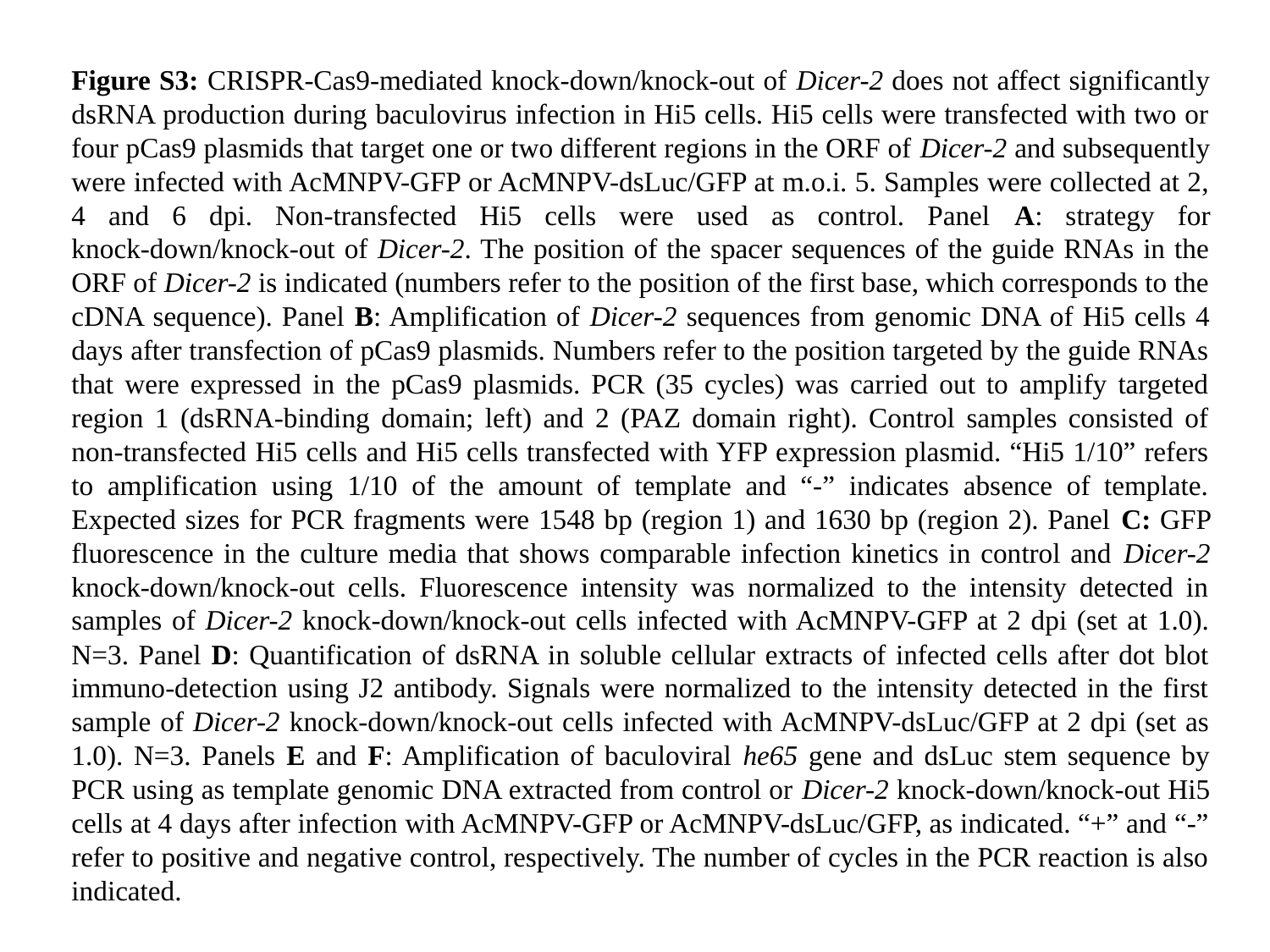

Figure S3: CRISPR-Cas9-mediated knock-down/knock-out of Dicer-2 does not affect significantly dsRNA production during baculovirus infection in Hi5 cells. Hi5 cells were transfected with two or four pCas9 plasmids that target one or two different regions in the ORF of Dicer-2 and subsequently were infected with AcMNPV-GFP or AcMNPV-dsLuc/GFP at m.o.i. 5. Samples were collected at 2, 4 and 6 dpi. Non-transfected Hi5 cells were used as control. Panel A: strategy for knock-down/knock-out of Dicer-2. The position of the spacer sequences of the guide RNAs in the ORF of Dicer-2 is indicated (numbers refer to the position of the first base, which corresponds to the cDNA sequence). Panel B: Amplification of Dicer-2 sequences from genomic DNA of Hi5 cells 4 days after transfection of pCas9 plasmids. Numbers refer to the position targeted by the guide RNAs that were expressed in the pCas9 plasmids. PCR (35 cycles) was carried out to amplify targeted region 1 (dsRNA-binding domain; left) and 2 (PAZ domain right). Control samples consisted of non-transfected Hi5 cells and Hi5 cells transfected with YFP expression plasmid. “Hi5 1/10” refers to amplification using 1/10 of the amount of template and “-” indicates absence of template. Expected sizes for PCR fragments were 1548 bp (region 1) and 1630 bp (region 2). Panel C: GFP fluorescence in the culture media that shows comparable infection kinetics in control and Dicer-2 knock-down/knock-out cells. Fluorescence intensity was normalized to the intensity detected in samples of Dicer-2 knock-down/knock-out cells infected with AcMNPV-GFP at 2 dpi (set at 1.0). N=3. Panel D: Quantification of dsRNA in soluble cellular extracts of infected cells after dot blot immuno-detection using J2 antibody. Signals were normalized to the intensity detected in the first sample of Dicer-2 knock-down/knock-out cells infected with AcMNPV-dsLuc/GFP at 2 dpi (set as 1.0). N=3. Panels E and F: Amplification of baculoviral he65 gene and dsLuc stem sequence by PCR using as template genomic DNA extracted from control or Dicer-2 knock-down/knock-out Hi5 cells at 4 days after infection with AcMNPV-GFP or AcMNPV-dsLuc/GFP, as indicated. “+” and “-” refer to positive and negative control, respectively. The number of cycles in the PCR reaction is also indicated.

## Slide 5
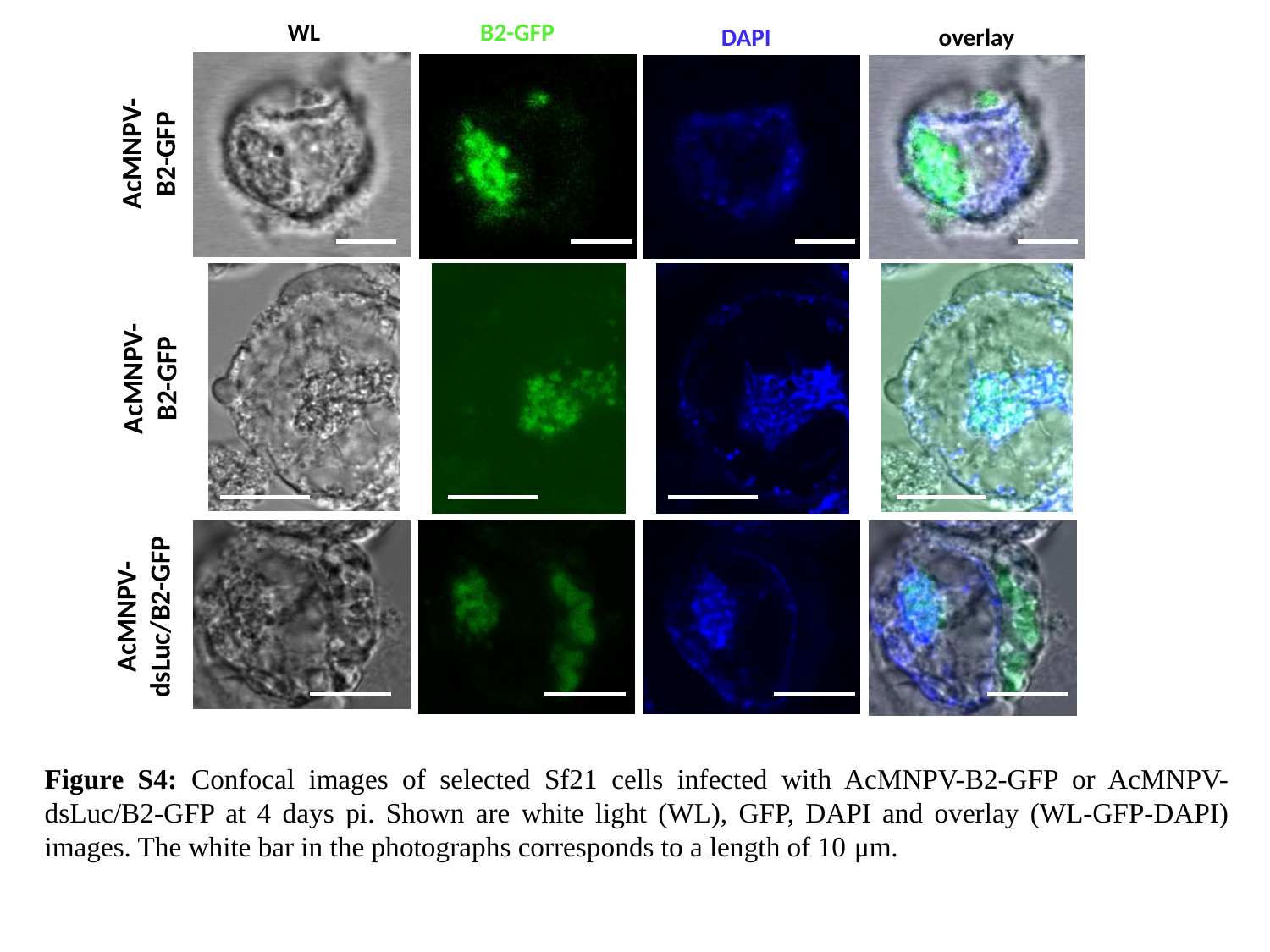

WL
B2-GFP
overlay
DAPI
AcMNPV-
B2-GFP
AcMNPV-
B2-GFP
AcMNPV-
dsLuc/B2-GFP
Figure S4: Confocal images of selected Sf21 cells infected with AcMNPV-B2-GFP or AcMNPV-dsLuc/B2-GFP at 4 days pi. Shown are white light (WL), GFP, DAPI and overlay (WL-GFP-DAPI) images. The white bar in the photographs corresponds to a length of 10 μm.

## Slide 6
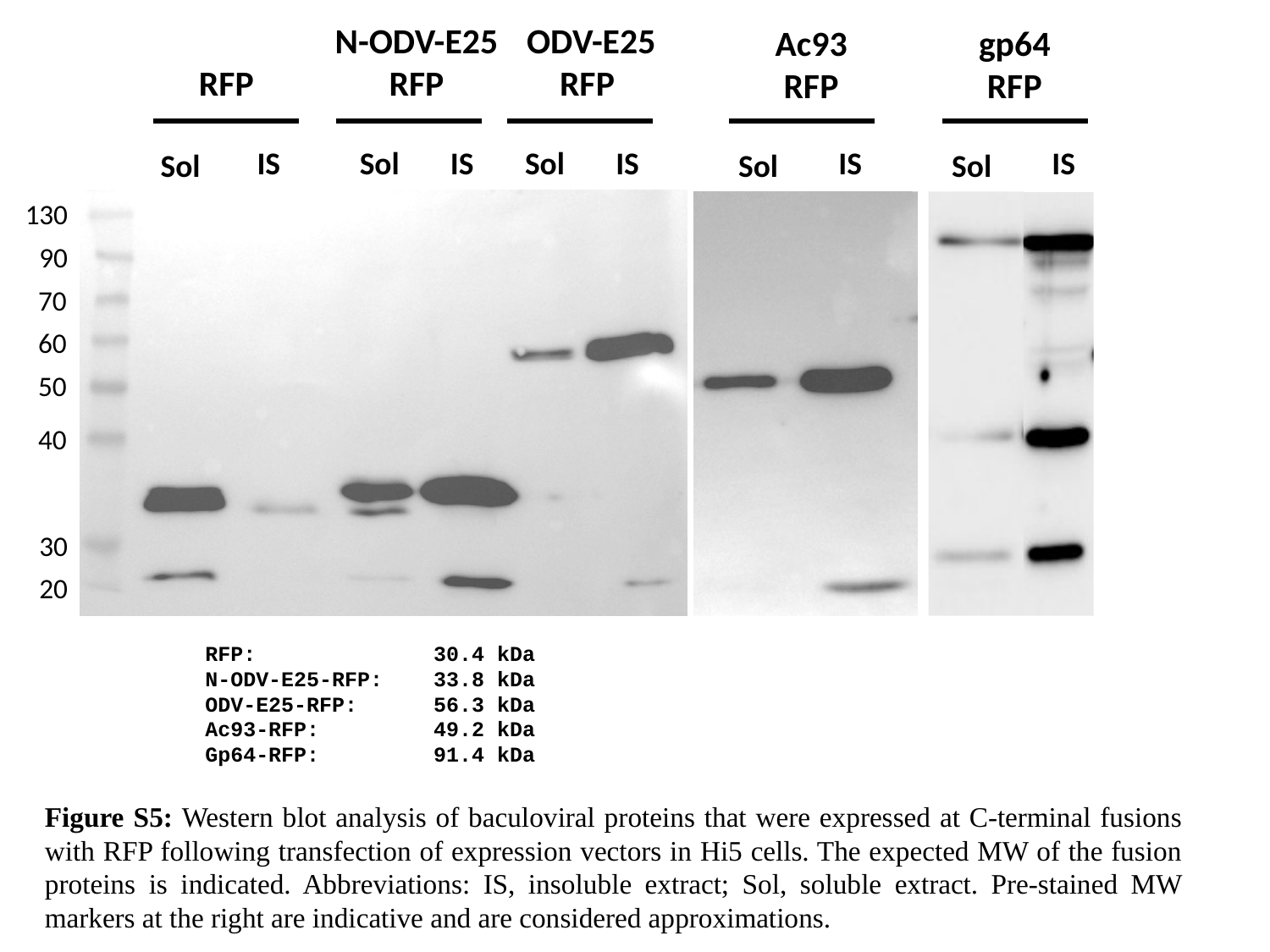

N-ODV-E25
RFP
 ODV-E25
RFP
Ac93
RFP
gp64
RFP
RFP
IS
Sol
IS
Sol
IS
Sol
IS
IS
Sol
Sol
130
90
70
60
50
40
30
20
RFP: 30.4 kDa
N-ODV-E25-RFP: 33.8 kDa
ODV-E25-RFP: 56.3 kDa
Ac93-RFP: 49.2 kDa
Gp64-RFP: 91.4 kDa
Figure S5: Western blot analysis of baculoviral proteins that were expressed at C-terminal fusions with RFP following transfection of expression vectors in Hi5 cells. The expected MW of the fusion proteins is indicated. Abbreviations: IS, insoluble extract; Sol, soluble extract. Pre-stained MW markers at the right are indicative and are considered approximations.

## Slide 7
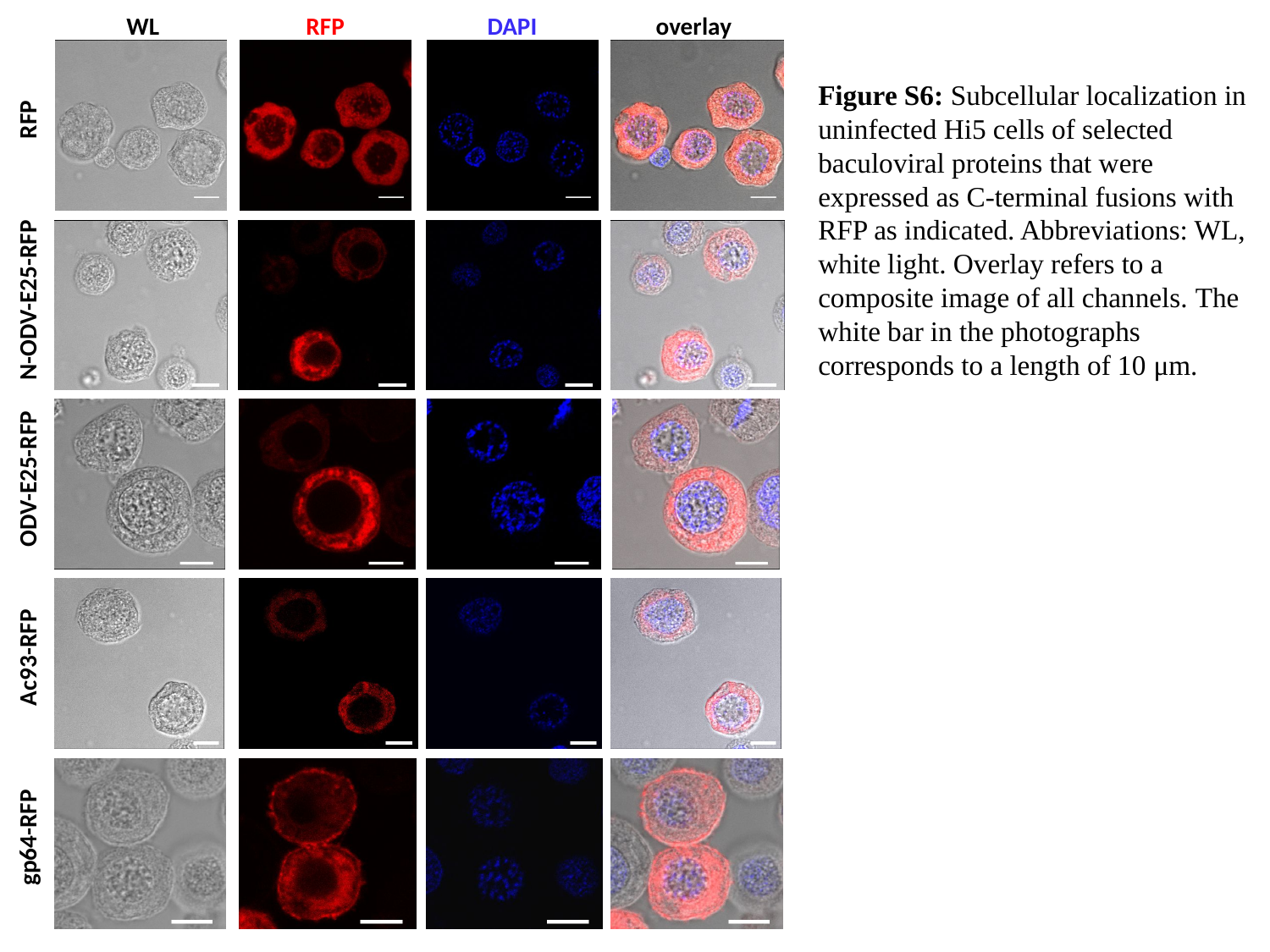

WL
RFP
DAPI
overlay
RFP
Figure S6: Subcellular localization in uninfected Hi5 cells of selected baculoviral proteins that were expressed as C-terminal fusions with RFP as indicated. Abbreviations: WL, white light. Overlay refers to a composite image of all channels. The white bar in the photographs corresponds to a length of 10 μm.
N-ODV-E25-RFP
ODV-E25-RFP
Ac93-RFP
gp64-RFP

## Slide 8
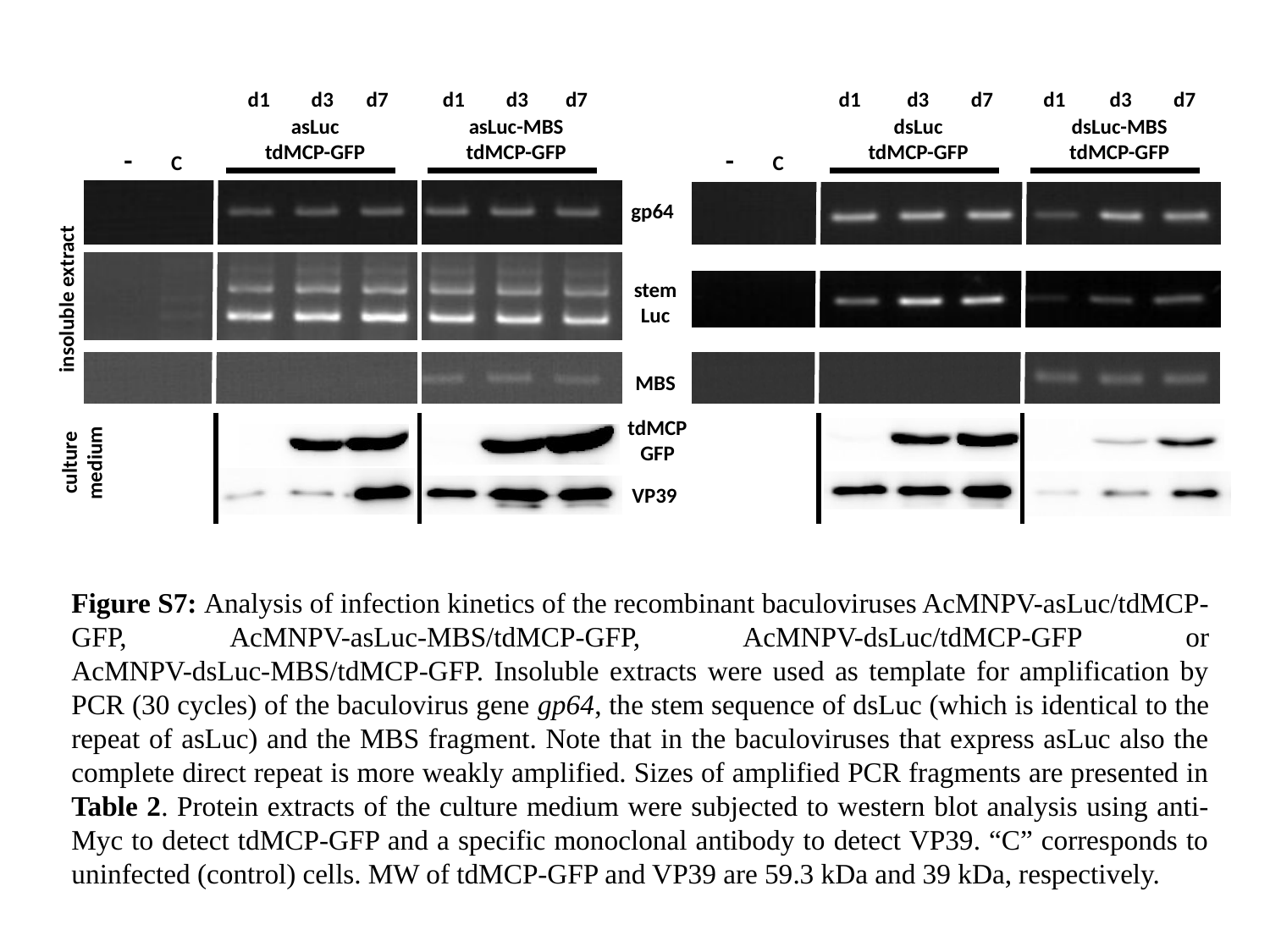

d1
d3
d7
d1
d3
d7
d1
d3
d7
d1
d3
d7
asLuc
tdMCP-GFP
asLuc-MBS
tdMCP-GFP
dsLuc
tdMCP-GFP
dsLuc-MBS
tdMCP-GFP
-
-
C
C
gp64
stem
Luc
MBS
tdMCP
GFP
culture
medium
VP39
insoluble extract
Figure S7: Analysis of infection kinetics of the recombinant baculoviruses AcMNPV-asLuc/tdMCP-GFP, AcMNPV-asLuc-MBS/tdMCP-GFP, AcMNPV-dsLuc/tdMCP-GFP or AcMNPV-dsLuc-MBS/tdMCP-GFP. Insoluble extracts were used as template for amplification by PCR (30 cycles) of the baculovirus gene gp64, the stem sequence of dsLuc (which is identical to the repeat of asLuc) and the MBS fragment. Note that in the baculoviruses that express asLuc also the complete direct repeat is more weakly amplified. Sizes of amplified PCR fragments are presented in Table 2. Protein extracts of the culture medium were subjected to western blot analysis using anti-Myc to detect tdMCP-GFP and a specific monoclonal antibody to detect VP39. “C” corresponds to uninfected (control) cells. MW of tdMCP-GFP and VP39 are 59.3 kDa and 39 kDa, respectively.

## Slide 9
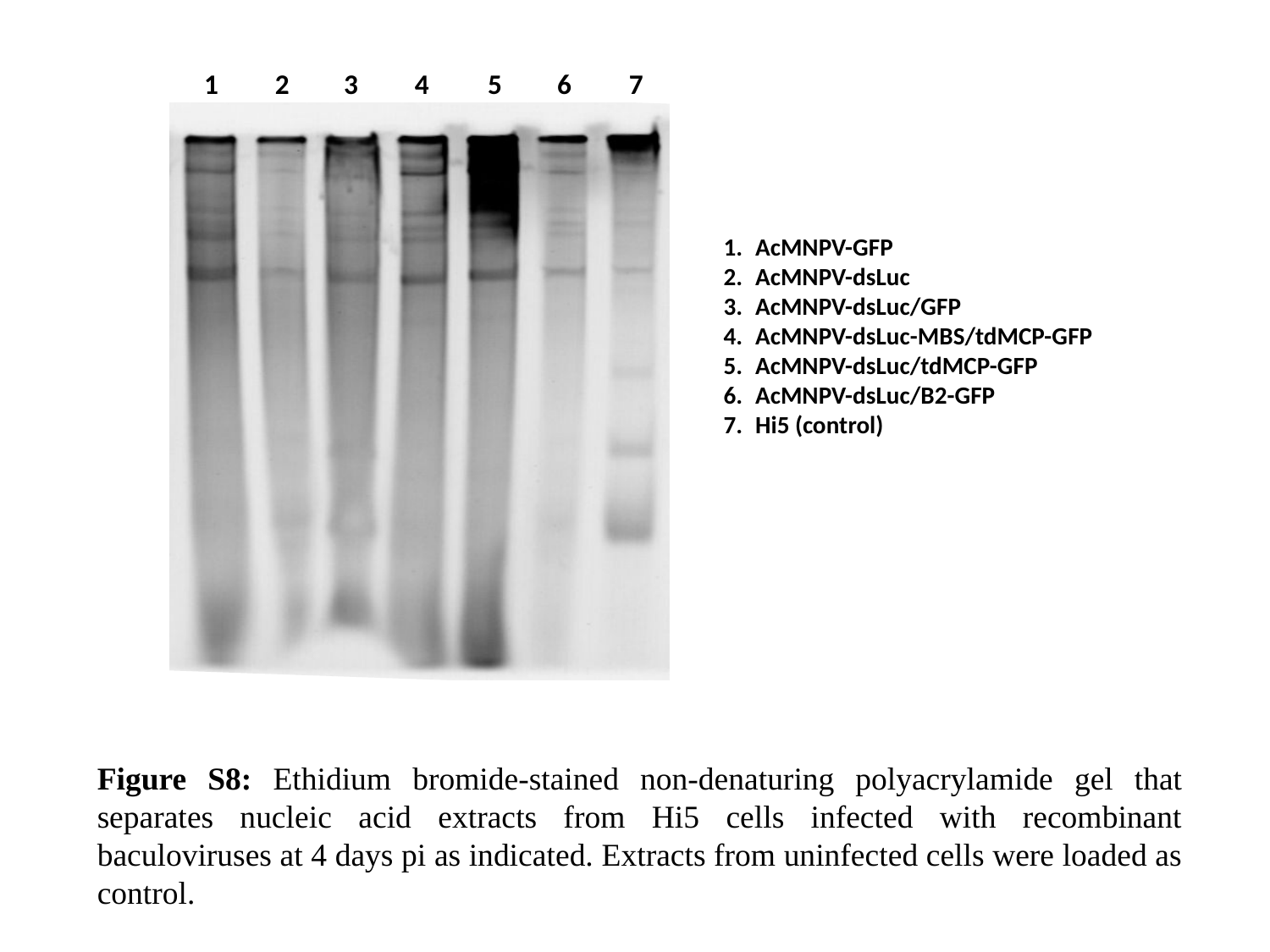

1
2
3
4
5
6
7
AcMNPV-GFP
AcMNPV-dsLuc
AcMNPV-dsLuc/GFP
AcMNPV-dsLuc-MBS/tdMCP-GFP
AcMNPV-dsLuc/tdMCP-GFP
AcMNPV-dsLuc/B2-GFP
Hi5 (control)
Figure S8: Ethidium bromide-stained non-denaturing polyacrylamide gel that separates nucleic acid extracts from Hi5 cells infected with recombinant baculoviruses at 4 days pi as indicated. Extracts from uninfected cells were loaded as control.

## Slide 10
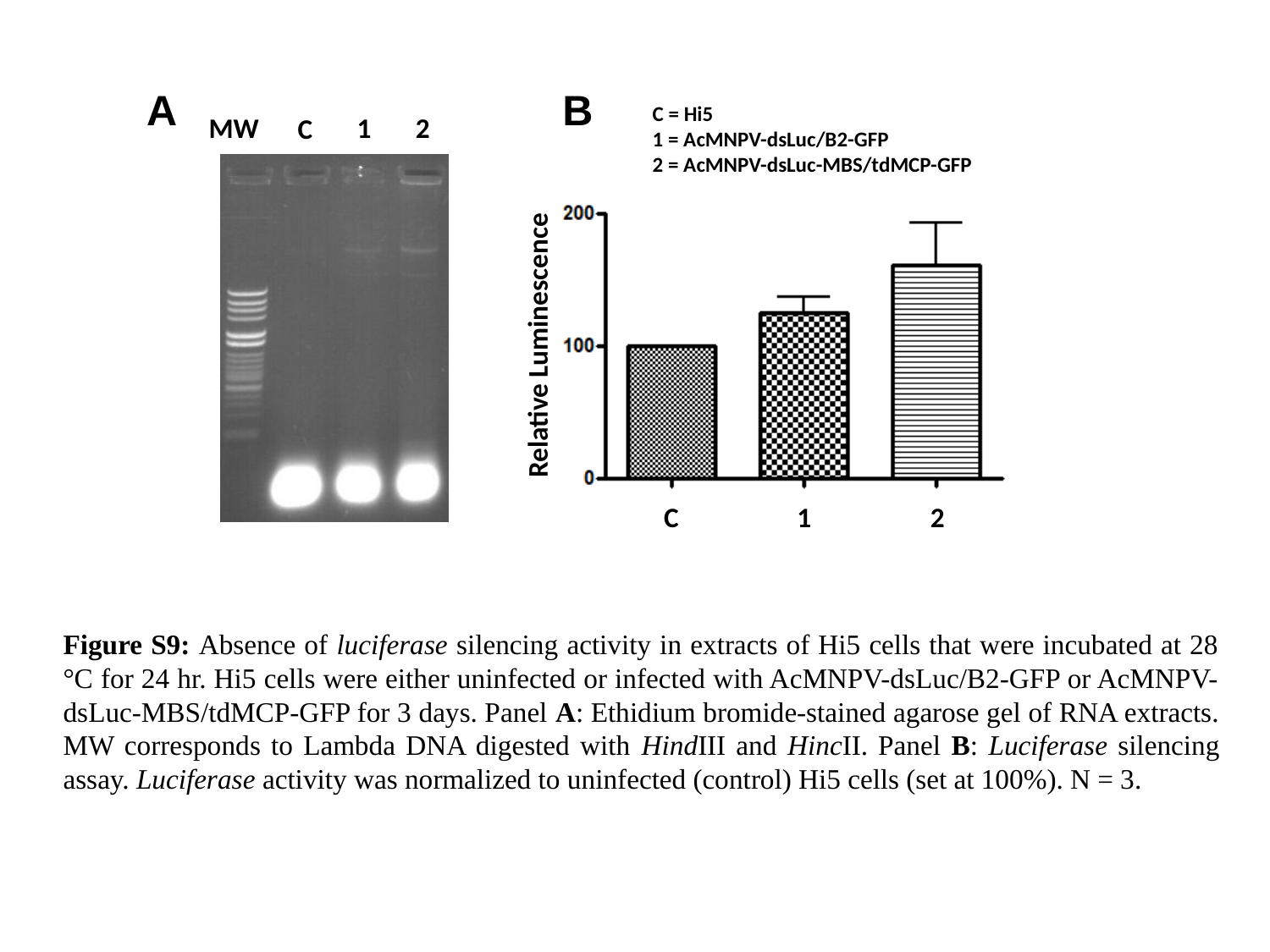

B
A
MW
1
2
C
C
1
2
C = Hi5
1 = AcMNPV-dsLuc/B2-GFP
2 = AcMNPV-dsLuc-MBS/tdMCP-GFP
Relative Luminescence
Figure S9: Absence of luciferase silencing activity in extracts of Hi5 cells that were incubated at 28 °C for 24 hr. Hi5 cells were either uninfected or infected with AcMNPV-dsLuc/B2-GFP or AcMNPV-dsLuc-MBS/tdMCP-GFP for 3 days. Panel A: Ethidium bromide-stained agarose gel of RNA extracts. MW corresponds to Lambda DNA digested with HindIII and HincII. Panel B: Luciferase silencing assay. Luciferase activity was normalized to uninfected (control) Hi5 cells (set at 100%). N = 3.
